# Quantifying submicrometer atmospheric aerosol chemical composition using nanoelectromechanical Fourier transform infrared spectroscopy

**DOI:** 10.1126/sciadv.aeb2254

**Published:** 2026-04-22

**Authors:** Mihnea Surdu, Radiance Calmer, Jelena Timarac-Popović, Tatjana Penn, Niklas Luhmann, Johannes Hiesberger, Veljko Vukićević, Erine Alvino Démolis, Lionel Favre, Berkay Dönmez, Hajrudin Bešić, Kostas Kanellopulos, Silvan Schmid, Josiane P. Lafleur, Satoshi Takahama, Julia Schmale

**Affiliations:** ^1^ Extreme Environments Research Laboratory, École Polytechnique Fédérale de Lausanne, Sion 1951, Switzerland.; ^2^ Invisible-Light Labs GmbH, Vienna 1040, Austria.; ^3^ Institute of Sensor and Actuator Systems, TU Wien, Vienna 1040, Austria.; ^4^ Laboratory of Atmospheric Processes and their Impacts, École Polytechnique Fédérale de Lausanne, Lausanne 1015, Switzerland.; ^5^ Laboratory of Environmental Spectrochemistry, École Polytechnique Fédérale de Lausanne, Lausanne 1015, Switzerland.

## Abstract

Atmospheric aerosols have major impacts on climate, air quality, and health, yet their chemical composition remains an analytical challenge due to their small size and low mass. We present an approach for aerosol chemical analysis combining nanoelectromechanical systems and Fourier transform infrared spectroscopy (NEMS-FTIR), offering simultaneous quantification of organic functional groups, ammonium sulfate, and equivalent black carbon with orders-of-magnitude lower detection limits over existing methods. The miniature, lightweight design allows for deployment on airborne platforms. Field demonstrations show that capturing distinct chemical compositions at the surface and aloft is possible, and they highlight the capacity of NEMS-FTIR for high-sensitivity, high–time-resolution aerosol analysis, revealing short-term variations in chemical composition previously inaccessible to conventional methods. NEMS-FTIR is therefore expected to advance the study of climate-relevant particles across different environments, from polluted urban to pristine polar. Beyond atmospheric science, this technique offers broader potential in nanoparticle analysis where mass constraints or time resolution is critical.

## Teaser

Ultrasensitive method using nanomechanical sensing and infrared spectroscopy enables chemical analysis of tiny airborne particles.

## INTRODUCTION

Atmospheric aerosols remain one of the largest uncertainties in our current understanding of climate change (*1*) and also have major impacts on air quality and human health (*2*, *3*). Upwards of 50% of cloud condensation nuclei, aerosols that seed cloud formation, originate from gas-phase nucleation and grow by condensation of low-volatility gases (*4*, *5*). Characterizing the chemical composition of these small particles is therefore essential for constraining how aerosols influence cloud microphysics and, in turn, Earth’s radiative balance (*6*, *7*). Equally important, ultrafine particles (<100 nm) pose substantial health risks due to their ability to penetrate deep into the respiratory and circulatory systems (*8*, *9*). However, due to their small size and low mass, chemical characterization of airborne aerosols remains an analytical challenge. Furthermore, aerosol composition may differ substantially between the surface and higher altitudes, where clouds form. This is particularly relevant in polar or mountainous regions with stable boundary layers (*10*). Capturing aerosol composition in situ at cloud elevations is critical for understanding climate-relevant processes.

Mass spectrometry has enabled substantial progress in this area. Instruments such as the aerosol mass spectrometer (AMS) provide real-time, quantitative data on submicron particles (*11*–*13*) and advanced our understanding of aerosol sources and composition globally (*14*–*16*). Nevertheless, the AMS does not efficiently transmit the smallest particles and relies on thermal vaporization and hard ionization, obscuring molecular structure (*17*, *18*). More recently, soft ionization techniques (*19*–*23*) have allowed for improved molecular-level characterization of ultrafine particles (*24*–*27*), but these systems remain large, expensive, and technically demanding, limiting their deployment in remote or mobile platforms.

Optical spectroscopy offers a complementary approach. Fourier transform infrared spectroscopy (FTIR) provides chemically specific information on functional groups (FGs) in complex aerosol mixtures (*28*–*31*), offering insight into volatility, growth mechanisms, and health impacts (*32*–*36*). Yet FTIR is typically performed offline using aerosols sampled on Teflon [polytetrafluoroethylene (PTFE)] filters (PTFE-FTIR), which limits time resolution and sensitivity, especially in low-mass environments e.g., 50 to 300 ng m^−3^ in the central Arctic summer (*37*), where extended sampling over days to weeks can obscure short-lived processes like particle formation or episodic transport. Other offline methods using gas or liquid chromatography coupled to mass spectrometry are also affected by this limitation (*38*, *39*).

Here, we present an analytical approach that combines nanoelectromechanical systems with FTIR (NEMS-FTIR), enabling chemical characterization of small (<300 nm) aerosol particles with substantially reduced sampling times, down to hours or even minutes. In this method, NEMS resonators act simultaneously as both substrate and detector for photothermal absorption spectroscopy, allowing for highly sensitive measurements of particle composition. The NEMS-FTIR sampling platform is miniature and lightweight, making it well suited for deployment in field campaigns and airborne measurements where instrument size and weight are critical constraints. NEMS-based infrared spectroscopy (IR) using, e.g., quantum cascade lasers, has previously demonstrated unprecedented sensitivity for targeted analysis of low-abundance species, such as in the analysis of pharmaceuticals, explosives, and aerosolized chemical standards (*40*–*45*). In this work, we expand the limited wavelength range of quantum cascade lasers to the analysis of the mid-infrared region typically used for aerosol spectroscopy by coupling a nanomechanical infrared analyzer (EMILIE, Invisible-Light-Labs GmbH, Austria) to FTIR. We show the capability of NEMS-FTIR to simultaneously quantify key aerosol components in environmental samples in the low nanogram range, such as organic FGs, ammonium sulfate (AS), and equivalent black carbon (eBC).

## RESULTS

### Aerosol collection onto NEMS resonators

**Fig. 1.**
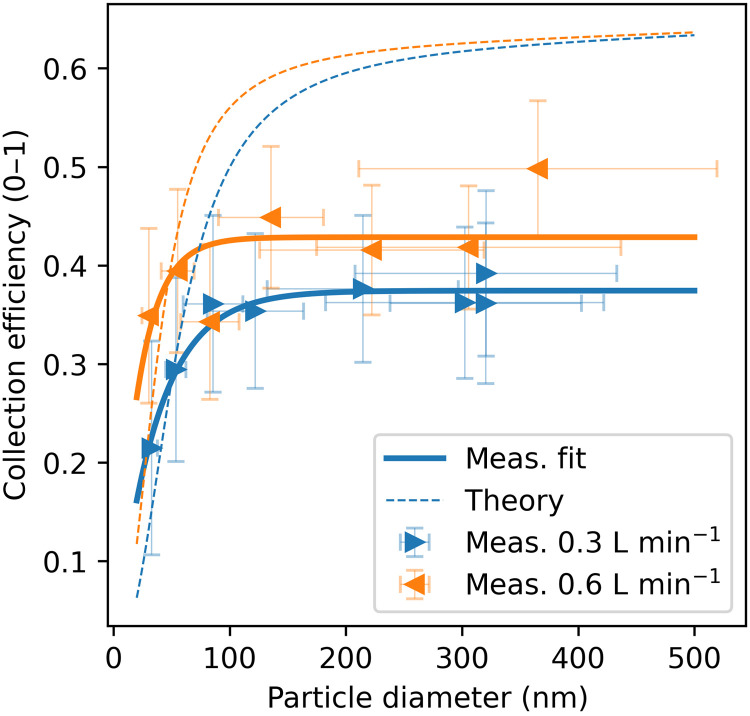
Size-dependent mass collection efficiency. Solid lines are exponential fits through the experimentally obtained markers for each flow rate (blue for 0.3 liters min^−1^ and orange for 0.6 liters min^−1^). The experimental markers are placed at the volumetric mean diameter on the *x* axis with error bars representing their SD. The *y*-axis error bars are calculated from SD of particle volume from multiple scanning mobility particle sizer (SMPS) scans. The theoretical efficiency curve is calculated as by Kurek *et al.* (*42*) and contains the effects of diffusion, impaction, and interception. Exponential curves were fit to the measurements, resulting in collection efficiencies *CE*(*D*_p_) of 0.374 * [1 − *e*^(−*D*p/35.432)^] and 0.429 * [1 – *e*^(−*D*p/20.614)^] for flow rates of 0.3 and 0.6 liters min^−1^, respectively. L, liters.

To quantify NEMS-FTIR spectra against particle sizing and counting methods, the particle diameter (*D*_p_)–dependent mass collection efficiency [*CE*(*D*_p_)] was determined. Airborne particulates are collected onto the NEMS resonators through the processes of impaction, interception, and diffusion. Experimentally, *CE*(*D*_p_) was determined for flow rates of 0.3 and 0.6 liters min^−1^ in the size range of 30 to 500 nm, using dry (solid) AS particles (Fig. 1). Collection efficiency increases with particle size, plateauing at around 40% above 100 nm. Experiments with wet (liquid) AS particles show an increased *CE*(*D*_p_) of around 55% at 150 nm (fig. S1), indicating that particle phase affects collection. The difference between solid and liquid particle *CE*(*D*_p_) is likely due to particle bounce, which reduces adhesion for solid particles but is less substantial for liquid aerosols. Theoretical calculations of *CE*(*D*_p_) (*42*), which do not account for bounce, more closely match the liquid particle measurements, supporting this interpretation. For typical mixed-phase ambient samples, uncertainties are limited to <19% (see Materials and Methods), indicating that our NEMS resonators can reliably collect submicron aerosols across a range of sizes and phase states.

### Quantitative evaluation of AS spectra

A comparison of NEMS-FTIR spectra to reference optical properties can be made by obtaining the decadic linear attenuation coefficient $\alpha_{10}\left(\tilde{\upsilon }\right)$ from the measured absorbance $A\left(\tilde{\upsilon }\right)$, assuming that the particles create an absorbing film with an effective thickness $d_{\text{eff}}$

**Fig. 2.**
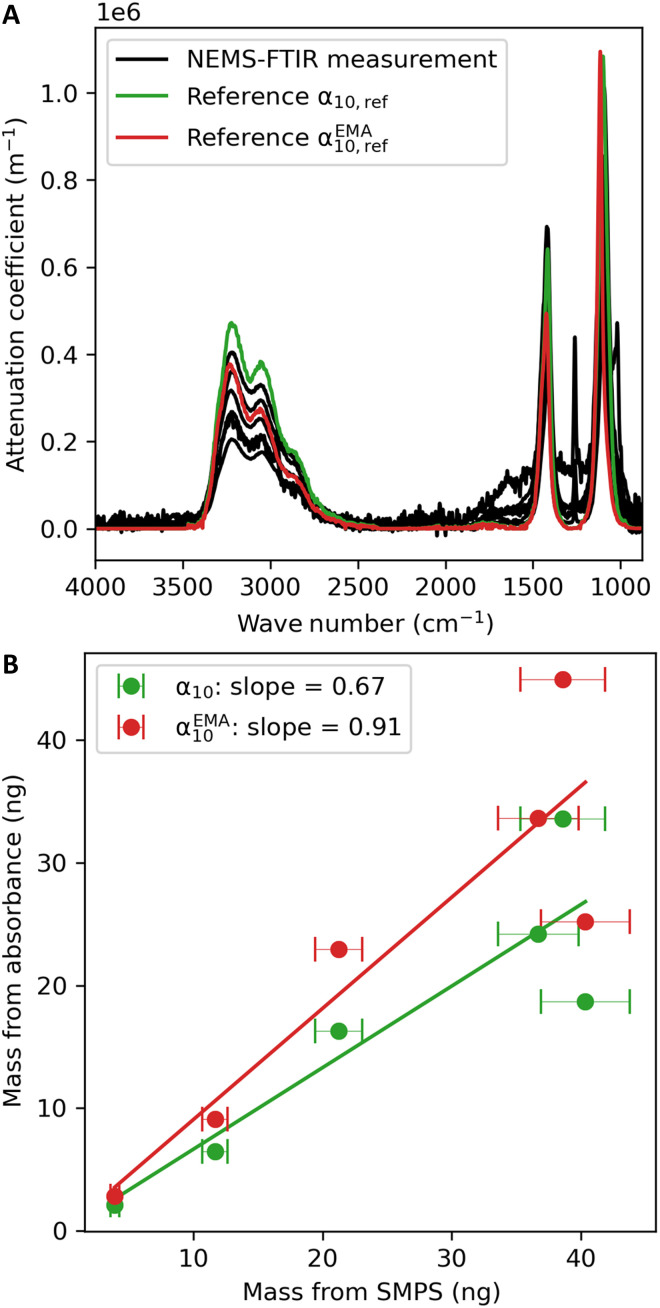
Quantitative evaluation of AS spectra. (**A**) A comparison of the experimentally determined decadic attenuation coefficients from NEMS-FTIR spectra of AS with the reference literature values (*46*). A sparse particle film is assumed by $\alpha_{10}\left(\tilde{\upsilon }\right)$, while $\alpha_{10}^{\left(\text{EMA}\right)}\left(\tilde{\upsilon }\right)$ assumes the sample forms a homogeneous film (see the Supplementary Materials). The peak at around 1260 cm^**−**1^ in some measurements is due to contamination from the silicone elastomer sticky-gel carrier box that some samples were stored in before analysis. (**B**) A comparison of the mass loadings calculated from SMPS measurements with those optically obtained from NEMS-FTIR absorbance with the reference literature attenuation coefficients in (A).

A(υ~)=α10(υ~) deff=α10(υ~)mcollectedSSρ (1)

where ρ is the density of the analyte, *m*_collected_ is the collected particle mass, and 𝑆_S_ is the sample area. Two different formulations of the attenuation coefficients were used for comparison: $\alpha_{10}\left(\tilde{\upsilon }\right)$, assuming that particles are sparsely distributed and not interacting optically, and $\alpha_{10}^{\left(\text{EMA}\right)}\left(\tilde{\upsilon }\right)$, which assumes the sample forms a homogenous film (see the Supplementary Materials). In Fig. 2A, the attenuation coefficients obtained from the NEMS-FTIR spectra of AS are compared with literature data for AS particles suspended in an aerosol flow tube (*46*).

The measured profiles have consistent peak positions of 1100 cm^−1^ ± 0.26% and 1419 cm^−1^ ± 0.10% for sulfate asymmetric stretch [ν_3_(SO_4_^2−^)] and ammonium asymmetric bend [ν_4_(NH_4_^+^)], respectively. This is in good agreement with the peak positions of $\alpha_{10}\left(\tilde{\upsilon }\right)$ from Earle *et al.* (*46*) of 1100 and 1417 cm^−1^ for ν_3_(SO_4_^2−^) and ν_4_(NH_4_^+^), respectively. Alternatively, the peak positions of ν_3_(SO_4_^2−^) and ν_4_(NH_4_^+^) in $\alpha_{10}^{\left(\text{EMA}\right)}\left(\tilde{\upsilon }\right)$ calculated from Earle *et al.* (*46*) are 1115 and 1423 cm^−1^, respectively, resulting in a small but noteworthy difference from the measurements for the sulfate peak (fig. S2). Generally, the spectral profiles from NEMS-FTIR are consistent with those from published optical properties, suggesting that the method does not introduce spectroscopic artefacts in this case. Liquid water is not observed in NEMS-FTIR spectra of liquid AS particles, consistent with evaporation as the analysis is performed under vacuum (see Supplementary Text S1 and fig. S3).

Using Eq. 1 and scaling literature attenuation coefficients (*46*) to the NEMS-FTIR absorbance in the region of 3000 to 3100 cm^−1^, the mass collected on the NEMS resonators can be obtained. Comparing this estimate to scanning mobility particle sizer (SMPS) measurements in Fig. 2B yields slopes of 0.67 and 0.91 depending on whether $\alpha_{10}\left(\tilde{\upsilon }\right)$ or $\alpha_{10}^{\left(\text{EMA}\right)}\left(\tilde{\upsilon }\right)$ was used. A Pearson’s correlation coefficient (*r*) = 0.9 is observed for both formulations, highlighting the linearity of NEMS-FTIR measurements and potential for quantification. Although the slope using $\alpha_{10}^{\left(\text{EMA}\right)}\left(\tilde{\upsilon }\right)$ is closer to unity, there is a slight peak shift for ν_3_(SO_4_^2−^) when using this formulation (fig. S2), which was previously also observed for AS particles collected on ZnSe (*47*). Given that the variance in attenuation coefficients calculated from refractive indices obtained from different methods is on the order of 25 to 30% (*46*–*49*), the NEMS-FTIR values are in the same magnitude range as previous literature values. Uncertainty in the NEMS-FTIR values may arise from the conversion of signal to absorbance, particularly due to variations in sample deposition area and the resonator’s relative responsivity, which is highest at the center of the membrane and decreases toward the edges (see Materials and Methods). Overall, the comparison with previous literature on AS shows that the NEMS-FTIR spectra can be directly compared to those obtained from other infrared techniques, both in terms of spectral profile and magnitude, allowing for analyte quantification.

### Quantification of aerosol chemical composition

**Fig. 3.**
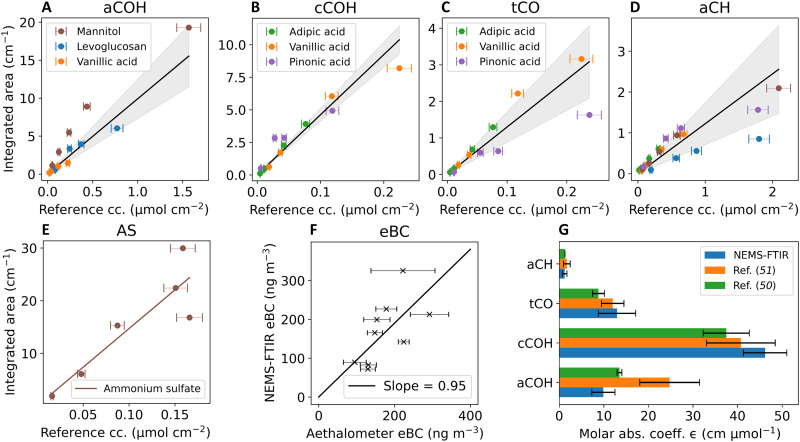
Calibrations for different chemical components. Peak area (integrated absorbance) is regressed against a reference concentration of the analyte (*y* intercept fixed at 0) for the FGs of aCOH (**A**), cCOH (**B**), tCO (**C**), aCH (**D**), and AS (**E**). Error bars represent the uncertainty of the collection efficiency used for calculating the reference concentration. Solid black lines in (A) to (D) show a combined fit wherein the slope is the weighted average, and the error is the SD of the individual compound slopes. The data are tabulated in the supplementary material (table S1). (**F**) A comparison of equivalent black carbon (eBC) measured by NEMS-FTIR and an aethalometer. Error bars represent the SD of the aethalometer measurements for the NEMS-FTIR sampling time. (**G**) The combined molar absorption coefficients for each FG compared with literature. cc., concentration.

In this work, organic FGs [alcohol C─OH (aCOH), carboxylic C─OH (cCOH), total carbonyl C═O (tCO), and alkane C─H (aCH)], AS, and eBC are quantified. Using the Beer-Lambert relationship, for an absorbing group *i* (here, an FG or AS) that absorbs over wave numbers $\tilde{\upsilon }$, the integrated absorbance A_i_^*^ (per centimeters) can be related to the areal density Γ (moles per square centimeter) (*50*)

$$A_{i}^{*}=\int A\left(\tilde{\upsilon }\right)\;d\tilde{\upsilon }=\epsilon_{i}\Gamma =\epsilon_{i}\frac{m_{\text{collected},i}}{S_{s}M_{i}}$$ (2)

where *M* is the molar mass. The areal density can be thought of as the moles of analyte per filter collection area, and ϵ is the integrated molar absorption coefficient (centimeters per mole). Details regarding the wave number regions used for sequential apportioning of the spectra into different FGs, AS, and eBC are provided in Materials and Methods. By regressing the integrated absorbance from the NEMS-FTIR spectra against the reference areal densities calculated from the SMPS data, ϵ can be obtained for all FGs of interest as the slopes of the regression with no intercept (Fig. 3, A to E). We found a Pearson’s *r* > 0.9 between the integrated peak area and reference concentration for all standards analyzed in this work. Taking the weighted average of ϵ for individual compounds, an average ϵ for each FG was obtained (table S1). For eBC, the mass concentrations from NEMS-FTIR were validated against collocated measurements with an aethalometer (Fig. 3F and fig. S4).

As ϵ is an intrinsic property of the analyte, the values obtained from NEMS-FTIR can be compared to those from previous PTFE-FTIR studies (Fig. 3G). A good agreement is observed for the aCH, tCO, and cCOH FGs between NEMS-FTIR and previous literature (*50*, *51*). For aCOH, the variation in ϵ between the three studies is larger, with the NEMS-FTIR value being closer to the earlier study of Takahama *et al.* (*50*). The difference in aCOH ϵ for the PTFE studies could be due to the high PTFE interference in the aCOH region, leading to difficulties in baseline correction. Variations may also arise due to the different selection of analytes used by the three studies, as ϵ for each FG varies according to the specific chemical environment in which the bond is present (*52*).

### NEMS-FTIR limits of detection

**Fig. 4.**
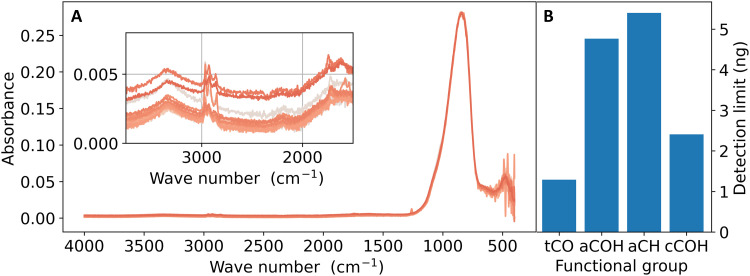
Blank spectra and estimated detection limits per FG. (**A**) Twenty-four normalized blank spectra with an inset of the wave number range above 1500 cm^**−**1^ used for estimating the detection limits. The spectra are colored in different shades of red for better visualization. The blank spectra feature an intrinsic large peak around 835 cm^**−**1^ from the SiN of the resonator membrane. Detection limits shown in (**B**) refer to the amount of FG deposited on the resonator.

Spectra from 24 empty NEMS resonators were used to estimate limits of detection (LODs; see Materials and Methods), which lie between 1.3 and 5.4 ng for the different organic FGs (Fig. 4). Previous PTFE-FTIR work reports detection limits in the low (1 to 6 μg) microgram range (*53*) for the FGs analyzed here, resulting in a roughly three orders of magnitude improvement in LODs for NEMS-FTIR. This improvement is likely largely due to the high sensitivity of the detector for photothermal absorption spectroscopy, as well as minimal substrate interference. A recent method was developed as an alternative to PTFE-FTIR to electrostatically collect aerosol particles onto ZnSe crystals, achieving an improved limit of quantification (LOQ) of 68 ng for AS (*47*) by removing substrate interference. While not directly comparable due to the different methodology for obtaining the LOD/LOQ, the current work brings roughly an order of magnitude lower detection limits when assuming the average organic FG LOD is applicable to AS.

Using the determined LODs to guide ambient sampling strategies, one can determine the minimum sampling time for a given mass concentration so that the mass loading on the resonator is above LOD for all FGs. We can consider here a hypothetical Arctic remote site with organic aerosol (OA) mass concentration of 0.5 μg m^−3^ and FG distribution as measured at Villum Research Station. This results in minimum sampling times of 98, 165, 70, and 183 min for tCO, aCOH, aCH, and cCOH to be above LOD, respectively, at a flow of 0.6 liters min^−1^. This is a remarkable reduction in sampling times by almost two orders of magnitude compared to the weekly sampling now being done for both PTFE-FTIR and mass spectrometric analysis of aerosols collected on filters in low-concentration environments (*28*, *54*). Similarly, for an urban location with an average OA mass concentration of 5 μg m^−3^, roughly 15 to 20 min of sampling ensure detection above LOD.

### Application of NEMS-FTIR to atmospheric samples

**Fig. 5.**
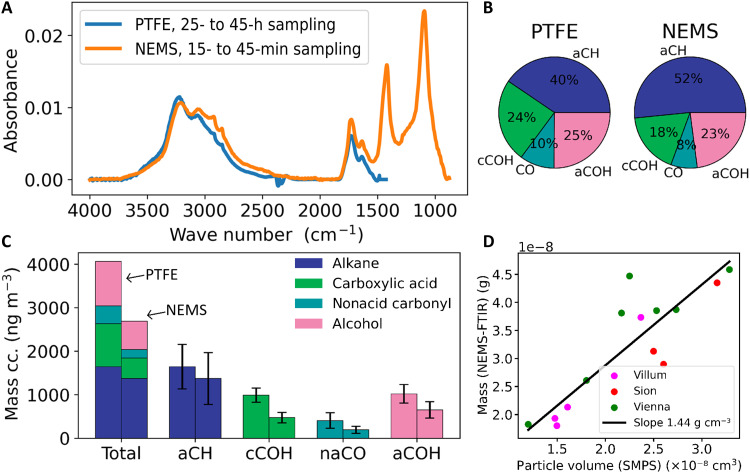
Comparison of organic FG quantification from NEMS resonators, PTFE filters, and SMPS mass. (**A**) Averaged PTFE (*n* = 2, sampling time of 25 to 45 hours at 9 liters min^**−**1^) and NEMS (*n* = 8, sampling time of 15 to 45 min at 0.7 liters min^**−**1^) spectra over the 3-day sampling period in Vienna. h, hour. (**B**) The relative FG distribution from the same samples. The ϵ values from the current study were used for the NEMS-FTIR data, while those from (*51*) were used for the PTFE data. (**C**) The quantified organic mass concentrations (cc.) for NEMS and PTFE substrates. Errors in (C) represent the weighted SD of absorption coefficients of different chemical standards used for calibration for PTFE. For NEMS, the error bars are the propagated error of baseline subtraction, collection efficiency and SD of ϵ of different chemical standards used in the calibration. (**D**) A comparison of the mass loadings estimated from NEMS-FTIR and SMPS total particle volume across ambient samples at all locations (*n* = 16), with the slope (Pearson’s *r* = 0.87) corresponding to the particle density. The mass from NEMS-FTIR is the sum of the organic FGs, AS, and black carbon.

To evaluate the suitability of NEMS-FTIR for ambient aerosol chemical characterization, measurements were taken at three sites: Sion (Switzerland, small town), Vienna (Austria, large city), and Villum Research Station (Greenland, remote Arctic observatory). In Vienna, eight NEMS resonators and two PTFE filters were sampled over 3 days with particles size selected <300 nm, leading to NEMS collection times ranging from 15 to 45 min and PTFE sampling over 25 to 45 hours (fig. S5). Figure 5 presents results averaged across this period. Direct comparison of mass concentrations and compositions is limited by the differing sampling durations and potential changes in aerosol populations. While PTFE filters provide a continuous average, NEMS-FTIR captures discrete snapshots that may not represent the entire period. In addition, PTFE scattering and C-F absorption in the region of 1400 to 1100 cm^−1^ obscures ammonium and sulfate peaks at 1419 and 1100 cm^−1^, respectively, which are visible in the NEMS-FTIR spectrum.

Despite this, the averaged spectra from both substrates show good qualitative agreement (Fig. 5A) and relative chemical compositions (Fig. 5B) are consistent within the uncertainties of each method. Total organic mass concentrations are similar (Fig. 5C), with NEMS values averaging 0.67 times those from PTFE. Given the difference in sampling times, it is difficult to ascertain with the current data whether this is a consistent underprediction by NEMS-FTIR. A possible factor could be the evaporation of semivolatiles for NEMS-FTIR as measurements are performed under vacuum. However, this is unlikely to fully explain the result as volatility-reducing (*33*) groups such as cCOH would be expected to evaporate more slowly than the bulk aerosol, yet cCOH is the only group showing a notable discrepancy.

Both techniques carry inherent uncertainties. Comparing FTIR-derived FG concentrations with those obtained from a reference method provides an estimate of the uncertainty associated with FTIR spectral analysis. In PTFE-FTIR, uncertainties arise from the peak fitting procedure, variation in absorption coefficients of standard compounds and baselining of the PTFE spectral signature. Previous PTFE-FTIR work reports mean errors of 8 to 33% for the quantification of different FGs, calculated for chemical standards as $\frac{1}{N}\sum_{i=1}^{N}|x_{r,i}-x_{p,i}|$ where *x*_r_ is the reference (gravimetric) concentration and *x*_p_ is the predicted concentration from the FTIR analysis of *N* measurements. For NEMS-FTIR, uncertainties from peak fitting and absorption coefficient variability are similarly present. Applying the same error analysis yields mean errors of 9.5 to 31% across different FGs (fig. S6), which also incorporate the uncertainty in collection efficiency applied to SMPS-derived reference concentrations. Additional uncertainties specific to NEMS-FTIR analysis of ambient samples arise from baseline subtraction (<25%, fig. S7) and particle bounce (<19%, fig. S2). Further errors for NEMS-FTIR may also arise due to uncertainties in the aerosol deposition onto the membrane. Here, we have assumed that the sample is evenly distributed on the perforated part of the NEMS resonator in order to convert the NEMS response to absorbance (see Materials and Methods). Due to the good agreement of NEMS absorbance with both the literature optical properties of AS and the literature ϵ values observed in this work, this additional uncertainty is likely small (<30%). The magnitude of the NEMS-FTIR uncertainties is comparable to those of established aerosol quantification methods, such as the AMS, which carries a conservative uncertainty estimate of around 38% (*55*). In practice, the discussed uncertainties are more likely to introduce a small systematic bias in NEMS-FTIR data rather than substantial random scatter, if the samples are collected and analyzed in a consistent manner. This has been observed in previous PTFE-FTIR work (*51*, *53*, *56*), where comparison to collocated ion chromatography, thermal/optical reflectance, and AMS measurements shows good correlations in terms of mass concentrations, albeit with a small systematic bias in the actual magnitudes.

Overall, the total mass calculated from NEMS-FTIR (as the sum of all FGs, AS, and eBC) is well correlated with the total SMPS particle volume collected onto the resonators (Pearson’s *r* = 0.87, Fig. 5D) across all three study locations. The regression slope of 1.44 g cm^−3^ represents the combined aerosol density. This aligns well with the reported densities of the main aerosol components, oxidized OA (*57*), and AS (around 1.3 and 1.77 g cm^−3^, respectively), as well as densities measured in the field at different locations (*58*–*60*).

**Fig. 6.**
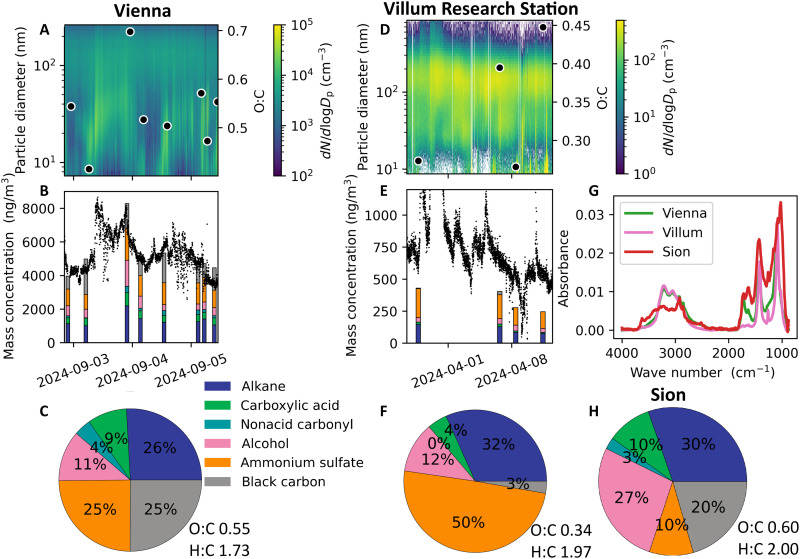
Case studies of atmospheric aerosol chemical composition at three locations. (**A** and **D**) Particle number size distributions and oxygen-to-carbon ratio (O:C). (**B** and **E**) Mass concentrations estimated from the SMPS measurements and quantified FG distributions from NEMS-FTIR. The SMPS mass is obtained using a density of 1.44 g cm^−**3**^. (**G**) Average NEMS-FTIR spectra at each location. The average relative FG chemical composition for each location is also shown in (**C**), (**F**), and (**H**).

Figure 6 presents case studies that highlight the high–time-resolution sampling enabled by NEMS-FTIR. The total mass concentrations from NEMS-FTIR follow those from the particle number size distribution, and the elevated concentration on 3 September is well captured by NEMS-FTIR. During this day, nucleation mode particles grow to ~100 nm. At the end of the growth event, the oxygen-to-carbon ratio (O:C) estimated from NEMS-FTIR (*61*) is considerably higher than before the event and the average over the studied period. This supports the role of more oxidized organics in ultrafine particle growth due to their lower volatility (*62*), observed here as increased abundance of aCOH and cCOH. Such processes would be difficult to observe with PTFE substrates, where information is smeared across the entire collection period. NEMS-FTIR data from Villum Research Station further demonstrate the ability to chemically characterize aerosols with short sampling durations (1.5 to 2 hours) even at low mass concentrations (0.5 μg m^−3^; Fig. 6, D to F).

Relative FG distributions differ across the three locations and sampling periods (Fig. 6, C, F, and H). AS contributes most at Villum Research Station (April 2024, 50%) and least in Sion (August 2024, 10%), consistent with high sulfate-to-organic ratios identified across the Arctic (annual mean, 0.4 to 0.7) and linked in winter to Arctic haze and transport from Eurasia (*54*). Conversely, lower AS fractions observed in Vienna and Sion align with previous findings from continental Europe (*15*, *63*). Higher eBC concentrations observed in Vienna and Sion reflect the urban character of these sites, while the remote Villum station exhibits significantly lower eBC levels.

In terms of the organic components, OA in Vienna and particularly Sion is more oxidized than that at Villum, as seen in the O:C, likely reflecting different sources and aging. AMS studies have shown high contributions of oxidized secondary OA (SOA) during summer from the atmospheric oxidation of biogenic vapors (*15*, *16*, *63*). The O:C values in Vienna (0.55) and Sion (0.60) lie within the AMS-derived range for semivolatile and low-volatile oxygenated OA [0.41 to 0.81; (*16*)], typically linked to SOA in summer. FG distributions show more alcohols in Sion and more carboxylic acids in Vienna, with minor contributions from nonacid carbonyls at both sites. While alcohols have been associated with marine sources (*30*, *64*) or biomass-burning emissions (*65*), however, these are unlikely for the current sampling periods and locations. Alternatively, literature has shown biogenic SOA to be rich in alcohol and peroxide FGs upon formation, with particle-phase processing converting these FGs to carboxylic acids and esters over time (*32*, *66*). Thus, it is plausible that both sites are influenced by biogenic SOA, with Sion reflecting fresher emissions from nearby forests and agriculture and Vienna showing more aged SOA. In contrast, OA sources at the end of winter in the Arctic are linked to primary organics (O:C of ~0.2), likely from gas flaring, and highly aged aerosol (O:C of ~0.8) from long-range Eurasian transport (*67*). The higher aCH fraction at Villum likely reflects contributions from fresh hydrocarbons, while elevated cCOH and aCOH suggest aged Arctic haze, yielding an intermediate O:C of 0.34.

Across all sites, the hydrogen-to-carbon ratio (H:C) estimated from NEMS-FTIR is rather high, closer to AMS values reported for fresh hydrocarbons (H:C of 1.92) than for more oxidized OA (H:C of 1.68 to 1.32) (*68*). A known positive offset (+0.2) in H:C for PTFE-FTIR relative to AMS has been previously reported for wood-burning aerosols, likely related to FTIR-uncharacterized aromatic and alkene carbon atoms, which may also affect the data from this work (*56*).

### Vertically resolved aerosol composition from NEMS-FTIR measurements

**Fig. 7.**
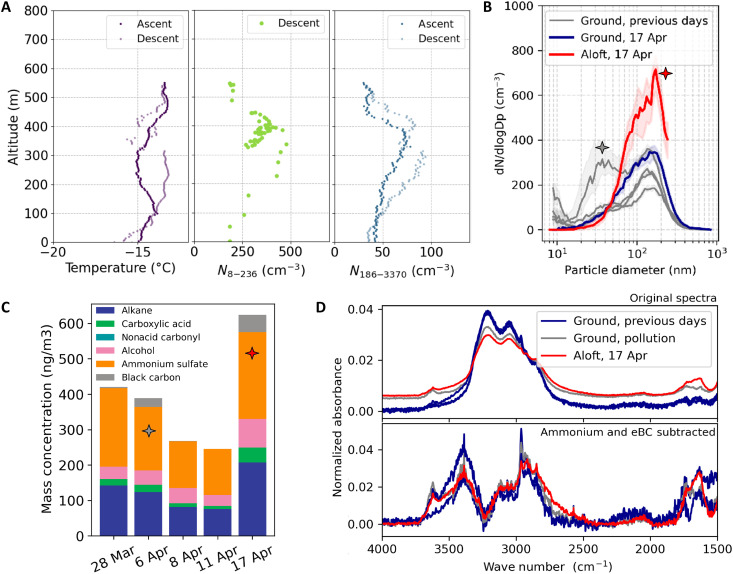
Vertical measurements of aerosols and their chemical composition at Villum Research Station. (**A**) Vertical profiles of temperature and total particle number concentrations for diameters of 8 to 236 nm and 186 to 3370 nm during ascent and descent on 17 April 2024. The NEMS-FTIR flight sample was taken while hovering at around 400 m altitude on 17 April 2024. (**B**) Median particle number size distributions during the sampling times of the NEMS resonators in (C) and (D). The gray curves are data from the ground SMPS, while the red curve is data from the flight instrument at altitudes of >200 m. Although no NEMS-FTIR sample was taken at the ground during the time of the flight, the blue curve represents the size distribution at the ground for comparison. The shaded areas denote the 25th to 75th percentile range. The gray star indicates a NEMS-FTIR sample influenced by local pollution, and the red star indicates a sample collected in flight. (**C**) The chemical composition from NEMS-FTIR. (**D**) Normalized (Euclidean norm) NEMS-FTIR absorbance before and after ammonium and eBC subtraction.

The miniaturized design of the NEMS resonators enables highly portable sampling, allowing for deployment on airborne platforms such as drones or tethered balloons. To explore the vertical distribution of aerosols and their chemical composition, we sampled NEMS resonators aboard a helikite tethered balloon at Villum Research Station (*69*). During the campaign, frequent strong surface-based temperature inversions are observed, typical of Arctic winter and spring, resulting in distinct aerosol populations at the surface and aloft (Fig. 7A and fig. S8).

At the surface, aerosol particle number size distributions feature a pronounced accumulation mode, consistent with Arctic haze (Fig. 7B) (*70*). Occasionally, Aitken mode particles produced from local pollution sources such as vehicle exhaust are also observed. Correspondingly, NEMS-FTIR measurements showed enhanced eBC concentrations (~25 ng m^−3^) in the surface sample influenced by local pollution compared to background ground-level conditions.

Above the inversion, a similar accumulation mode to the surface was observed, although particle number concentrations were elevated. The NEMS-FTIR spectra showed compositional differences relative to the surface layer while also preserving key similarities (Fig. 7, C and D). NEMS-FTIR derived mass concentrations were higher aloft, consistent with the particle size distribution measurements. Equivalent BC concentrations were also enhanced aloft compared to days when surface measurements were not influenced by local pollution. The OA component (as the sum of aCH, aCOH, cCOH, and nonacid carbonyl FGs) was enriched above the temperature inversion, yielding an organic-to-sulfate ratio of 1.35 compared to 0.95 (±0.07) at the surface. Despite this enrichment, the normalized NEMS-FTIR spectra (after subtracting AS and eBC contributions) reveal that the organic component aloft is compositionally very similar to that at the surface (Fig. 7D).

These observations can be linked to known transport patterns in the Arctic, where pollution is commonly lifted up into the troposphere at lower latitudes, followed by northward transport and gradual descent in the Arctic along stable isentropic layers (*71*). Here, 7-day air mass back trajectories identify transport from Arctic and subarctic Canada both aloft and at the surface (fig. S9), for all the NEMS resonator sampling durations. Examination of the trajectory altitudes shows that these air masses gradually descended from high altitudes (>2 km) over the previous 7 days before reaching our site (fig. S10), suggesting that any boundary-layer emissions were entrained at lower latitudes more than a week before sampling. Due to the strongly stratified atmosphere in the wintertime Arctic, pollution layers found at higher altitudes are typically associated with transport into the region along higher isentropic pathways that originate in warmer, lower-latitude areas (*72*). This supports the hypothesis that air masses sampled aloft, at higher potential temperatures, likely originate from warmer lower latitudes than those arriving at the ground level. Although beyond the 7-day simulated air mass travel time in this work, this would imply that the NEMS-FTIR measurements at the ground represent aged emissions from higher-latitude North America and those aloft from lower latitudes in North America. This mechanism may explain the observed enhancement of organics and eBC over AS in the flight sample as they can originate from lower-latitude emission sources, as also observed by the study of Willis *et al.* (*73*). Lower-latitude source regions contain comparatively higher anthropogenic organic (using CO as a proxy) and black carbon emissions than SO_2_ (fig. S11) and are also richer in vegetation that emits biogenic gases.

Given this variability in aerosol source regions and therefore aging timescales, the observed strong similarity in the OA component at the ground and aloft is unexpected. This similarity suggests that the OA is already sufficiently aged by the time of measurement, such that differences in the original emissions are no longer detectable. The modest O:C of 0.34 could be explained by primary particles (e.g., from gas flaring) mixed with aged anthropogenic pollution (i.e., Arctic haze), as observed at Villum Research Station in prior work (*67*) and discussed in the previous section. This aligns with observations of gas flaring over Canada (fig. S11) and previous studies observing markers of gas flaring in the Arctic after a 10-day-long aging from the source of emission, suggesting that gas flaring primary emissions do not rapidly oxidize further (*74*). Therefore, OA has likely already reached its maximum aging before arrival at Villum Research Station for both surface and flight samples and does not readily undergo further chemical transformation, at least at the low temperatures involved.

## DISCUSSION

The performance advances of NEMS-FTIR, including ~1000-fold lower detection limits and expanded spectral coverage relative to PTFE-FTIR, have important implications for atmospheric research. Reduced sampling times enable investigation of chemical changes on timescales relevant to particle formation, growth, and transformation, processes often missed by longer-term integrated sampling. In this work, we used a preimpactor (<300 nm) to target the smallest submicron particles, demonstrating that NEMS-FTIR can provide size-resolved chemical information. The high sensitivity of the method could compensate for reduced collection efficiency for particles of <70 nm, extending chemical characterization toward the ultrafine regime and enabling quantitative analysis of health-relevant yet low-mass aerosols with an appropriate size-selection method.

The compact size, low weight, and high sensitivity of NEMS-FTIR allow for chemical composition measurements throughout the vertical column, addressing a current gap in instrumentation that has been repeatedly identified in polar atmospheric research (*75*–*77*). Compared to conventional studies using aircraft and complex instrumentation (e.g., AMS), NEMS sampling aboard a tethered balloon or drone offers lower-cost deployment and substantially simpler logistics. This can allow for more systematic measurements and allows additional observations in regions and seasons where data are lacking, for example, during winter months in polar environments. By providing vertical chemical profiles, including speciation of organics into FGs, NEMS-FTIR measurements can be used to examine the performance of regional chemical-aerosol models and improve the representation of aerosol-cloud interactions where observational data are scarce.

Applying this capability in our Arctic field campaign revealed that aerosol chemical composition can differ substantially between the surface and higher altitudes, particularly under stable boundary layer conditions. Because cloud formation occurs above the temperature inversion, such vertical heterogeneity directly affects aerosol-cloud interactions and emphasizes that measurements at the surface may not be representative. Accurate modeling of cloud properties is important as cloud radiative forcing remains a major uncertainty in Arctic climate projections (*1*). In this low-aerosol environment, cloud microphysical properties are sensitive not only to aerosol number concentrations but also to their chemical and physical characteristics (*75*). Concretely, the higher relative abundance of eBC and organics aloft in our observations results in aerosols with lower hygroscopicity than at the surface, which has implications on the size and number of cloud droplets. If models were to extrapolate the hygroscopicity from surface observations for the entire vertical column, then it would result in an overestimation of cloud longwave radiation. Such biases can propagate, potentially leading to errors in modeled Arctic energy balance and sea-ice melt, an important climate tipping point.

## MATERIALS AND METHODS

The chemical composition of aerosols is measured using NEMS resonators, which act both as substrate and detector. Aerosols are first sampled on NEMS resonators inside a modified commercially available multichannel sampler. Thereafter, NEMS resonators are transferred to the analysis chamber coupled to an FTIR light source to perform photothermal absorption spectroscopy. When analytes on the surface of the NEMS chip absorb IR light, they undergo local heating, leading to thermal expansion and a reduction in tensile stress. This results in a measured frequency shift of the NEMS chip, which is proportional to the amount of absorbed power. A schematic diagram including a visualization of the process is provided in fig. S12. The resulting absorption spectra are processed (see fig. S13) and analyzed to quantify FGs present in the aerosol sampled.

### NEMS resonators

The NEMS resonators in this work were fabricated in the cleanroom, either in-house or commercially (EMILIE chips, Invisible-Light Labs GmbH, Austria). They consist of a 50-nm thin square silicon nitride membrane with an area of 1 mm^2^ deposited on silicon wafers. A circular area of 600 μm in diameter in the center of the membrane contains perforations of 6 ± 0.5 μm diameter, resulting in a perforated area of ~0.108 mm^2^ and a porosity of 0.37.

### NEMS sampling setup

An eight-channel filter sampler (FILT, Brechtel Manufacturing Inc., USA) is used for aerosol sampling. This allows for remote-controlled and automated sampling of multiple resonators. The original filter holders were exchanged by custom-made stainless steel holders specifically designed to house the NEMS resonators (Aerosol Flow Adapter, Invisible-Light-Labs GmbH, Austria). The sampling flow rate chosen was the maximum flow rate, which did not cause the NEMS resonators to burst due to the pressure drop across the resonator, which, for the configuration in this study, was between 0.5 and 1 liters min^−1^. After sampling, resonators were either immediately analyzed or stored in the freezer (−20°C) until further analysis.

### Setup for determining collection efficiency

AS was aerosolized using an aerosol nebulizer from a dilute distilled deionized water (DDW) solution and then dried using a silica gel dryer. The relative humidity was not measured; however, it is likely low (<30%) due to the silica-gel dryer. Particles were neutralized with a soft x-ray charger (Model 9002, Brechtel Manufacturing Inc., USA) and size selected using the differential mobility analyzer (DMA) of a miniaturized scanning electrical mobility sizer (mSEMS; Brechtel Manufacturing Inc., USA). The particle size distribution of the size-selected particles was then measured with a different scanning electrical mobility sizer (SEMS; Brechtel Manufacturing Inc., USA). To determine the collection efficiency, a holder containing the NEMS resonator was periodically inserted upstream of this SEMS (directly downstream of its neutralizer). The size-dependent collection efficiency is then determined as the difference between the particle volume with and without the NEMS resonator in the flow path (effectively, the particle volume lost to the resonator), divided by the total particle volume without the resonator in the flow path. The flow rate through the NEMS resonator was controlled by the particle counter downstream and was kept constant at either 0.3 or 0.6 liters min^−1^ for two different sets of experiments.

Additional experiments were carried out with polydisperse AS aerosol, aerosolized from a DDW solution. Silica-gel dryers with different residence time were used to dry the particles to 40 and 12% RH, respectively. Without a drier, the RH was 90% immediately upstream of the NEMS sampling. An impactor was used to remove large droplets before sampling. The collection efficiency was measured as for the size-selected experiments above, using a SEMS.

The mean bias of using the collection efficiency calculated from the solid monodisperse particle experiments [$\mathrm{CE}{\left(D_{p}\right)}_{\text{solid}}$] to liquid samples was determined to be −19%, calculated as $\frac{1}{N}\sum_{i=1}^{N}\left[\mathrm{CE}{\left(D_{p}\right)}_{\text{solid},i}-\mathrm{CE}{\left(D_{p}\right)}_{\text{liquid},i}\right]$ for *N* measurements, where $\mathrm{CE}{\left(D_{p}\right)}_{\text{liquid}}$ was obtained from the AS polydisperse experiments at 40 and 90% RH (above efflorescence RH).

In this study, aerosols were mostly collected at flow rates of either 0.5 or 0.7 liters min^−1^, and $\mathrm{CE}{\left(D_{p}\right)}_{\text{solid}}$ at 0.6 liters min^−1^ was used, possibly introducing a bias of ±16%, given that efficiency scales linearly with aerosol velocity (*78*). The mass collected on the resonator (*m*_collected_) is linked to the ambient mass concentration (*c*_aerosol_) and *CE*(*D*_p_) as follows

$$m_{\text{collected}}=c_{\text{aerosol}}\;\times \;Q\;\times \;t\;\times \;\mathrm{CE}\left(D_{p}\right)$$ (3)

where *t* is the sampling time and *Q* is the sampling flow rate. The uncertainty in $\mathrm{CE}{\left(D_{p}\right)}_{\text{solid}}$ is estimated at 8.5% as the average error across all measured data points.

### Calibrations with chemical standards

Analyte standards in this work were dissolved in DDW and aerosolized using an aerosol nebulizer. The aerosol airstream was dried using a silica-gel dryer. Afterward, the flow was split into two branches: one for sampling aerosol onto the NEMS resonators and another for particle sizing and counting. Excess flow was discarded. Particle sizing and counting was performed by either a SEMS or an mSEMS. The mass of aerosol sampled on the chip is calculated using Eq. 3.

### Ambient sampling

Ambient aerosols were collected on the surface of NEMS resonators at two urban locations (Sion, Switzerland and Vienna, Austria) as well as one Arctic site (Villum Research Station, Station Nord, Greenland). Collocated particle sizing and counting was performed using an mSEMS for samples from Vienna and Sion, and a mobility particle size spectrometer, consisting of a medium Vienna-type DMA combined with a condensation particle counter (model TSI 3772), at Villum Research Station. While no preimpactor was used when sampling aerosols in Villum or Sion, sampling in Vienna occurred downstream of a PM 0.3 impactor (SKC, USA). Sample collection times for the NEMS resonators ranged between 15 and 120 min, depending on the measured aerosol mass concentration. For the Vienna ambient sample collection, aerosols were also simultaneously collected on PTFE filters (Teflon, 2.0 μm, 47 mm, Pall Corporation, USA) at a flow rate of ~9.3 liters min^−1^. In Sion, collocated eBC measurements were conducted with a Microaeth MA200 Aethalometer (AethLabs, USA) measuring the light attenuation at 880 nm, using a flow rate of 0.15 liters min^−1^, time resolution of 60 s, and DualSpot enabled. MA200 data were postprocessed with a six-point moving average. Previous work has shown excellent comparability of the MA200 with conventional aethalometers used for long-term monitoring (*79*).

### Tethered balloon measurements

Vertical measurements were performed at Villum Research Station using a helium-based tethered balloon helikite (Allsopp Helikites Ltd.). The payload is modular and can carry a variety of instruments (*69*). Here, an mSEMS was used for particle counting and sizing in the range of 8 to 236 nm and a portable optical particle sizer (Handix Scientific) for diameters 186 to 3370 nm. Temperature was measured using a SHT85 humidity and temperature sensor (Sensirion). The filter sampler [(FILT), Brechtel Manufacturing Inc., USA] used for ground-based sampling was also used to sample NEMS resonators in flight, with remote-controlled operation.

### NEMS-FTIR analysis setup

Analysis of the NEMS resonators was carried out using a commercially available nanomechanical infrared analyzer (EMILIE, Invisible-Light-Labs GmbH, Austria), under vacuum conditions (<10^−4^ mbar). The NEMS resonator was cooled down using an integrated Peltier element to a temperature of +5°C to reduce evaporative losses.

The resonance frequency of the NEMS resonators was tracked using a self-sustaining oscillator circuit (*80*, *81*) (PHILL, Invisible-Light Labs GmbH, Austria), which provided a voltage signal proportional to the measured frequency shift. This signal was delivered to the FTIR spectrometer (Vertex 70 or Invenio R, Bruker Optics, Germany) via the dedicated interface for external detectors (E550/A, Bruker Optics, Germany). NEMS-FTIR spectra were recorded using the step scan mode with a stabilization delay of 30 ms, 200 coadditions, and spectral resolution of 4 cm^−1^. The aperture was set to 6 mm.

PTFE filters were analyzed using a Bruker Vertex 80 FTIR instrument (Bruker Optics, Germany) equipped with an α deuterated lanthanum alanine-doped triglycine sulfate detector in transmission-mode with an aperture of 6 mm. A spectrum of the empty sample compartment taken just before the sample spectrum was used as the reference for conversion to absorbance. Sixty-four scans were taken for both the background and sample spectra.

### NEMS-FTIR signal conversion to absorbance

The spectral processing workflow is summarized in fig. S13, with visualizations of the spectral changes as they undergo processing. A Python script to apply the spectral processing steps described in this work is provided. More detail for the signal conversion to absorbance units is provided in a separate manuscript (*82*).

First, the power absorbed by the analyte $P_{\text{abs}}\left(\tilde{\upsilon }\right)$ as a function of wave number $\tilde{\upsilon }$ is normalized by the power spectrum of the FTIR spectrometer $P_{0}\left(\tilde{\upsilon }\right)$ to account for variations in light intensity as a function of wave number. Here, $P_{0}\left(\tilde{\upsilon }\right)$ was obtained using a NEMS resonator featuring a 5-nm platinum thin film linear absorber (EMILIE LIGHT chip, Invisible-Light Labs GmbH, Austria), which provides a nominal absorptance of 50% from near- to far-infrared (*83*). This gives a measure of the variations in source power as a function of wave number albeit not the true magnitude of the source power. The power-corrected spectra [$P_{\text{abs}}\left(\tilde{\upsilon }\right)/P_{0}\left(\tilde{\upsilon }\right)$] are then normalized such that the large Si─N peak normally found in the region of 835 cm^−1^ is equal to 1. The result of this normalization is termed here the NEMS signal $N\left(\tilde{\upsilon }\right)$

$$N\left(\tilde{\upsilon }\right)=\frac{P_{\text{abs}}\left(\tilde{\upsilon }\right)}{P_{0}\left(\tilde{\upsilon }\right)}/\frac{P_{\text{abs}}\left(835\;{\text{cm}}^{-1}\right)}{P_{0}\left(835\;{\text{cm}}^{-1}\right)}$$ (4)

Blank spectra obtained from empty NEMS resonators, which have also undergone the above processing steps, are now subtracted from the analyte spectra. For the samples stored in the freezer, an empty resonator was kept under identical conditions and used as a reference for subtraction. This approach aims to minimize the effect of vapor deposition onto the cold resonators as some frozen samples are affected by small amounts of alkane deposition.

The NEMS signal can be converted to absorptance, $\mathrm{Ab}\left(\tilde{\upsilon }\right)$, making use of the fact that the resonator is made of SiN which features a large intrinsic peak at 835 cm^−1^ that can serve as an internal standard. At 835 cm^−1^, N(SiN)=b×Ab(SiN) where *b* is a unitless scaling factor. Because N(SiN) is equal to one following the normalization, we can obtain *b* as the inverse of the absorptance of SiN [Ab(SiN)], which was determined from transmission-mode measurements of 12 empty NEMS resonators to be 0.21 ± 0.01.

For the conversion of the NEMS analyte signal into absorptance, additional factors need to be considered such that

$$N\left(\tilde{\upsilon }\right)=\frac{S_{S}}{S_{\text{IR}}}\;\times \;\gamma \;\times \;b\;\times \;\mathrm{Ab}\left(\tilde{\upsilon }\right)=\frac{S_{S}}{S_{\text{IR}}}\;\times \;\gamma \;\times \;\mathrm{Ab}\left(\tilde{\upsilon }\right)/\mathrm{Ab}\left(\text{SiN}\right)$$ (5)

where *S*_IR_ and *S*_S_ are the areas of the infrared beam and sample, respectively, and γ is a unitless correction factor for the thermal responsivity of the membrane. Effectively, $\frac{S_{S}}{S_{\text{IR}}}$ takes into account that the entire square NEMS resonator made of SiN is irradiated with infrared light, while the sample is deposited onto a subset of that area. The sample is assumed to be evenly deposited on the central perforated area of the resonators, such that $S_{S}$ is equal to the area of the circular perforated area minus the area of the perforations (0.292 − 0.108 = 0.184 mm^2^). The infrared beam area reaching the resonator for an FTIR aperture of 6 mm is a circle with diameter larger than the square NEMS resonator, such that $S_{\text{IR}}$ is the area of the square SiN resonator minus the area of the perforations (1 − 0.108 = 0.892 mm^2^). The thermal responsivity factor γ accounts for the responsivity of the resonator being higher in the center and decreasing in intensity toward the edges. For a circle with a radius of 300 μm corresponding to the sample deposition area under the assumption that the sample is evenly distributed on the central perforated part of the membrane, as seen from microscope images, γ was determined from finite element model simulations to be 1.57. This means the response is 1.57 times higher than for a sample evenly distributed over the entire square membrane.

Equation 5 can be reformulated to solve for absorptance, which can be converted to absorbance. Assuming sample scattering to be negligible, absorbance $A\left(\tilde{\upsilon }\right)$ is related to transmittance $T\left(\tilde{\upsilon }\right)$ and absorptance by Eq. 6. Following Eq. 5, for small values of absorptance, absorbance can be linearized using a first-order Taylor series such that

$$\begin{array}{c} A\left(\tilde{\upsilon }\right)={-\text{log}}_{10}\left[T\left(\tilde{\upsilon }\right)\right]=\\ {-\text{log}}_{10}\left[1-\mathrm{Ab}\left(\tilde{\upsilon }\right)\right]\approx \frac{\mathrm{Ab}\left(\tilde{\upsilon }\right)}{\text{ln}\left(10\right)}=N\left(\tilde{\upsilon }\right)\times \frac{\mathrm{Ab}\left(\mathrm{SiN}\right)}{\frac{S_{S}}{S_{\text{IR}}}\;\times \;\gamma \;\times \;\text{ln}\left(10\right)} \end{array}$$ (6)

Spectra were then truncated below 865 cm^−1^ as the FTIR light power rapidly decreases at lower wave numbers, leading to noisy spectra.

### Quantification and subtraction of black carbon

The majority of ambient samples featured a negatively sloping baseline as the wave number decreases from 4000 to 1000 cm^−1^, suggesting the presence of black carbon, whose tail end of the electronic transition stretches into the mid-infrared region (*84*, *85*). Therefore, the contribution of black carbon was obtained and subtracted as follows. Assuming a sparse particle film, the complex refractive index of diesel soot as a function of wave number [$\tilde{n}\left(\tilde{\upsilon }\right)$] was obtained from literature (*86*) and converted to the decadic linear attenuation coefficient [$\alpha_{10}\left(\tilde{\upsilon }\right)$], using the relation (see derivation in Supplementary Text S3)

$$\alpha_{10}\left(\tilde{\upsilon }\right)=\frac{6\pi \tilde{\upsilon }}{\text{ln}\left(10\right)}\text{Im}\left\{\frac{{\tilde{n}}^{2}\left(\tilde{\upsilon }\right)-{\tilde{n}}_{m}^{2}\left(\tilde{\upsilon }\right)}{{\tilde{n}}^{2}\left(\tilde{\upsilon }\right)+2{\tilde{n}}_{m}^{2}\left(\tilde{\upsilon }\right)}\right\}$$ (7)

with the complex refractive index of the medium ${\tilde{n}}_{m}\left(\tilde{\upsilon }\right)$. The collected nanoparticles are positioned on top of a 50-nm-thick suspended silicon nitride resonator. Due to its subwavelength thickness, the silicon nitride layer is optically thin and does not significantly affect the effective refractive index of the surrounding medium, as confirmed by finite element simulations (fig. S14). Therefore, the refractive index of the surrounding medium can be approximated by that of vacuum, i.e., ${\tilde{n}}_{m}=1.$ The formulation in Eq. 7 for the decadic linear attenuation coefficient is applicable for particles smaller than the wavelength of the probing light, where absorption is the dominant contributor to attenuation and scattering is negligible. Reference soot $\alpha_{10}\left(\tilde{\upsilon }\right)$ was scaled to the NEMS-FTIR spectra in the region between 4000 and 3796 cm^−1^ as previous literature shows good agreement between the integrated absorbance in that region and collocated elemental carbon measurements, as well as a lack of absorption from other compounds (*85*). Using Eq. 1, the collected eBC mass can be estimated. Here, a density of 1 g cm^−3^ was used, as an average of literature reported values of both fresh and aged soot (*87*). The scaled soot reference spectrum was subtracted, and subsequent processing was done on the soot-subtracted spectra. In the case of chemical standard spectra, the soot subtraction step was not carried out as no soot is expected to be present.

### Baseline correction

Following soot subtraction, the spectra still exhibited a residual baseline, indicating the need for additional correction. This likely stems from the diesel soot spectrum used for subtraction not being a perfect match for the black carbon deposited on the NEMS resonators. As soot from different sources (e.g., flame soot) can exhibit subtle spectral variations, the subtraction may have somewhat over- or undercorrected certain spectral regions. To preserve the soot signature, baseline correction was applied after soot subtraction. The baseline correction implemented here closely resembles the smoothing splines approach developed for infrared spectra of aerosols on PTFE filters (*88*). Briefly, the spectra are divided into two regions: region 1 between 4000 and 1820 cm^−1^ and region 2 between 2000 and 865 cm^−1^. The baseline for each region is obtained by minimizing the function

$$\sum_{i}^{N}w_{i}\left(y_{i}-z_{i}\right)+\lambda \int {\left[z^{\prime \prime }\left(x\right)\right]}^{2}\mathrm{dx}$$ (8)

where *y_i_* is the absorbance, *z_i_* is the estimated baseline, λ is a smoothing factor, and *w_i_* is the weighting for the *i*th wave number. The first term minimizes the distance between the baseline and the absorbance, while the second term is a regularization term controlling the smoothness of the baseline function. For the overlap of the two spectral regions, the mean absorbance was used. Weights were set to 0 for wave numbers where we expect analyte absorption and 1 in background regions. The bounds of the analyte and background regions of the spectra are determined according to the methods described in detail by (*88*). Unlike the PTFE filter spectra, which are truncated at 1500 cm^−1^, the spectra from the NEMS resonators in this work are truncated at 865 cm^−1^. Therefore, the lower bound of the analyte region in region 2 of the spectra was extended and determined as the wave number where the absorbance reaches its minimum between 1100 and 865 cm^−1^. The baseline minimization function was run iteratively for a range of λ, and the user is prompted to choose the best fit of λ for that particular spectrum based on visual inspection. An example of the choice of baseline and a sensitivity test for varying λ is provided in the Supplementary Materials and is used to estimate the errors associated with baseline correction (figs. S7 and S15). The baseline correction was also performed on chemical standard spectra for consistency, although the subtracted baselines were minimal for most standard spectra.

### Fitting of organic FGs and AS

Following baseline subtraction, absorption profiles specific to targeted FGs (line shapes) are fit to the spectra. In condensed phase spectra, absorption bands are broad due to shifts in vibrational frequencies induced by interactions with the surrounding molecular environment. We fit peaks in the FG region of the spectrum (4000 to 1500 cm^−1^), where characteristic absorption bands that correspond to specific chemical bonds appear. The peak fitting protocol developed for PTFE-FTIR [AIRSpec; (*89*)] is used here, with the addition of fitting for AS. Gaussian peaks are used for peak fitting, assuming that the absorption for a specific FG is due to the sum of many peaks from individual compounds. For PTFE-FTIR, sulfate is not commonly quantified because of the interference from the PTFE substrate itself in the fingerprint region of the spectra. In the case of the NEMS resonators, this interference is not present, which allows using this technique to additionally quantify AS. As for the soot fitting, the complex refractive index of AS is obtained from the literature (*46*) and converted to $\alpha_{10}\left(\tilde{\upsilon }\right)$ using Eq. 7. The line shapes for AS and cCOH overlap in the region around 3000 cm^−1^, prompting us to first fit the cCOH line shape as it has no overlapping absorption in the area of 2600 cm^−1^. The AS $\alpha_{10}\left(\tilde{\upsilon }\right)$ is subsequently scaled to the cCOH-subtracted spectra between 3100 and 3000 cm^−1^. Other organic FGs (aCOH, aCH, and tCO) are fit in AIRSpec according to the well-established methodology previously developed and validated for PTFE filters (*50*, *51*, *89*). For the PTFE filters analyzed in this work, spectra were first processed by subtracting the blank spectrum whose PTFE spectral signature is most similar to the analyte spectrum. Afterward, the baseline subtraction and peak fitting procedure detailed in (*88*) and (*51*) were performed in the AIRSpec package (*89*).

### Determination of LODs

Spectra from 24 empty NEMS resonators were used to estimate LODs. The blank spectra feature a large peak in the region of 830 to 840 cm^−1^ from the SiN of the resonator material itself. Some spectra also feature a peak at 1260 cm^−1^, resulting from cross-contamination by the silicone elastomer sticky-gel carrier box in which some chips were stored. Besides that, sharp alkane peaks in the region around 3000 cm^−1^ and a broad peak at around 3300 cm^−1^ are present in different abundances across the blank spectra. As there is no characteristic water peak at 1640 cm^−1^, we assign the peak at 3300 cm^−1^ to hydroxyl group and conclude the absence of liquid water condensation. These contaminant peaks are likely from two sources: (i) compounds on the walls of the analysis chamber may re-partition and deposit on the surface of the resonators during analysis as the chip is cooled below room temperature and (ii) airborne contaminants may also deposit on the resonator surface during storage between sampling and analysis. Experiments analyzing a blank NEMS resonator at temperatures from −5° to 45°C show only minor differences in the contaminant peaks, suggesting that they originate primarily from airborne contaminants deposited onto the resonator surface rather than condensation of organic vapors (fig. S16). After the heating steps to 45°C, we stepwise cooled the chamber back to 15°C. Analysis upon cooling the chamber shows additional contamination at lower temperatures, suggesting that condensation of organic vapors onto the colder resonator can occur if they have been allowed to volatilize in the analysis chamber.

The detection limits for each target FG were determined by fitting the line shapes used for aerosol analysis to the blank spectra. The LODs were then calculated as three times the SD of the peak area for each FG across the 24 blank spectra, using ϵ in table S1 for conversion to mass. It is likely that, in practice, these obtained LODs are an upper estimate for two reasons. First, the spectrum from a blank resonator stored under the same conditions and analyzed at a similar time (with comparable chamber contamination) was subtracted to reduce variation from contamination signatures. Moreover, the spectra used for LOD calculation are offset above the baseline by different amounts, leading to variance in the fitted peak areas. The systematic offsets are due to spectra being multiplied by a different value for normalization to the SiN peak, with spectra being acquired with a larger output range for converting the resonance frequency to voltage having a smaller SiN peak before normalization. The effect on the LODs should largely be cancelled out by the baseline-correction step when analyzing sampled spectra.

## Supplementary Materials

### This PDF file includes:

Table S1 Figs. S1 to S16 Supplementary Text S1 to S3 References
