# Supplementary material for: Quantifying submicrometer atmospheric aerosol chemical composition using nanoelectromechanical Fourier transform infrared spectroscopy
Source: Sci Adv. 2026 Apr 22;12(17):eaeb2254. doi: 10.1126/sciadv.aeb2254 (PMC13101882; doi:10.1126/sciadv.aeb2254)
Supplement: Supplementary file 1 — Table S1 Figs. S1 to S16 Supplementary Text S1 to S3 References [file sciadv.aeb2254_sm.pdf]

Science Advances
AAAS

# Supplementary Materials for

## Quantifying submicrometer atmospheric aerosol chemical composition using nanoelectromechanical Fourier transform infrared spectroscopy

Mihnea Surdu *et al.*

Corresponding author: Mihnea Surdu, mihnea.surdu@epfl.ch; Julia Schmale, julia.schmale@epfl.ch

*Sci. Adv.* **12**, eaeb2254 (2026)
DOI: 10.1126/sciadv.aeb2254

**This PDF file includes:**

Table S1
Figs. S1 to S16
Supplementary Text S1 to S3
References

**Table S1: Molar absorption coefficients ($\epsilon$) for each functional group and compound.** Errors correspond to the standard error of the linear fit in the case of single compounds, and the standard deviation of all individual compound $\epsilon$ in the case of the combined $\epsilon$.

| | Molar absorption coefficient $\epsilon$ (cm $\mu mol^{-1}$) | | | | |
|---|---|---|---|---|---|
| **Compound** | **aCOH** | **cCOH** | **tCO** | **aCH** | **AS** |
| Mannitol | 13 ± 1 | - | - | 1.06 ± 0.09 | - |
| Levoglucosan | 9 ± 1 | - | - | 0.52 ± 0.04 | - |
| Vanillic acid | 7.0 ± 0.5 | 40 ± 4 | 15 ± 1 | 1.49 ± 0.06 | - |
| Adipic acid | - | 52 ± 1 | 16 ± 0.3 | 2.1 ± 0.1 | - |
| Pinonic acid | - | 47 ± 8 | 7.1 ± 0.5 | 1.0 ± 0.2 | - |
| Ammonium sulfate | - | - | - | - | 147 ± 16 |
| **Combined** | 10 ± 3 | 46 ± 5 | 13 ± 4 | 1.2 ± 0.5 | 147 ± 16 |

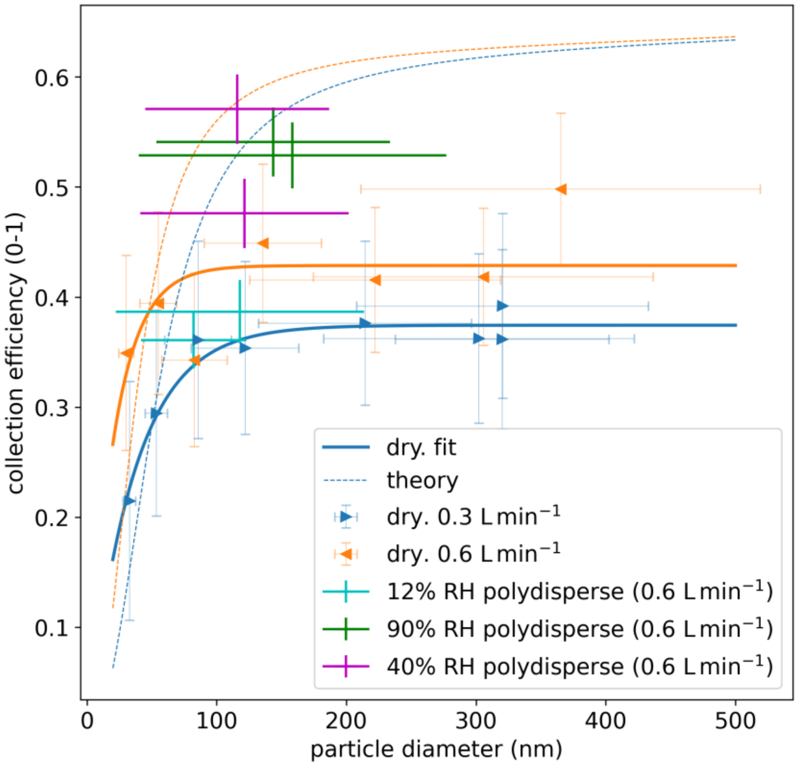


**Figure S1: Size-dependent mass collection efficiency.** Same as main text Figure 1, with the addition of polydisperse ammonium sulfate datapoints at 12, 40, and 90% RH.

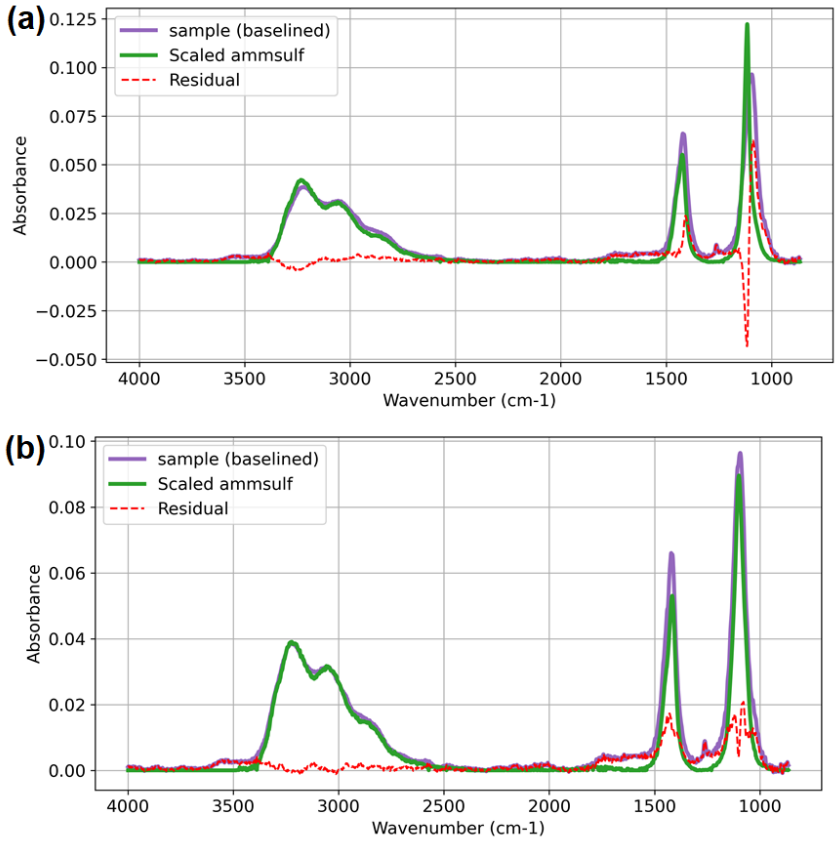


**Figure S2: Comparison of measured NEMS-FTIR ammonium sulfate spectra (purple) to reference attenuation coefficients.** The green curve corresponds to ammonium sulfate $\boldsymbol{\alpha}_{\mathbf{10}}^{(EMA)}(\tilde{\boldsymbol{v}})$ and $\boldsymbol{\alpha}_{(\mathbf{10})}(\tilde{\boldsymbol{v}})$ in panels (a) and (b), respectively (Earle et al., 2006).

## Text S1. Analysis of solid and liquid ammonium sulfate particles.

Ammonium sulfate particles were generated with an atomizer from a dilute solution. Silica-gel dryers with different residence time were used to dry the particles to 40% and 12% RH respectively. Without a drier, the RH was 90% immediately upstream of the NEMS sampling. An impactor was used to remove large droplets prior to sampling. Spectra of the ammonium sulfate particles are shown in Figure S3.

We do not observe absorbance due to liquid water (normally seen at 3500-3300 $cm^{-1}$ and around 1640 $cm^{-1}$) in any of the spectra. This is consistent with the water evaporating from the particles as the analysis is carried out under vacuum (< $10^{-4}$ mbar). However, we do observe a peak shift for the sulfate asymmetric stretch ($v_3$($SO4^{2-}$)) from around 1100 $cm^{-1}$ at 40% and 90% RH to roughly 1117 $cm^{-1}$ at 12% RH, similar to aerosol flow tube infrared spectroscopy studies. There is also a slight blue-shifting of the ammonium asymmetric bend around 1419 $cm^{-1}$ for the 12% RH sample (solid).

This observation suggests that at 12% RH (below efflorescence), the ammonium sulfate particles are solid. Although we did not observe liquid water absorbance, the peak position for the 40% and 90% RH (above efflorescence) ammonium sulfate particles is consistent

with literature peak positions of aqueous ammonium sulfate particles. This observation can be interpreted as the particle-bound water rapidly evaporating as the sample is put under vacuum, leaving behind a metastable liquid with just trace amounts of water. In contrast, for the 12% RH sample all of the water was removed in the long residence time in the silica gel dryer, resulting in a crystalline solid particle.

Overall, the phase state of the particles may give rise to slight peak shifts, although liquid water is not visible in NEMS-FTIR spectra as it evaporates under vacuum. Given the differences in peak location for each of the thousands of molecules likely present in ambient samples, due to the bonds being in different chemical environments as part of different molecules, the phase state does not impart additional uncertainty. This is due to the fact that the spectral peaks are fit using broad Gaussian functions, which inherently account for such variability.

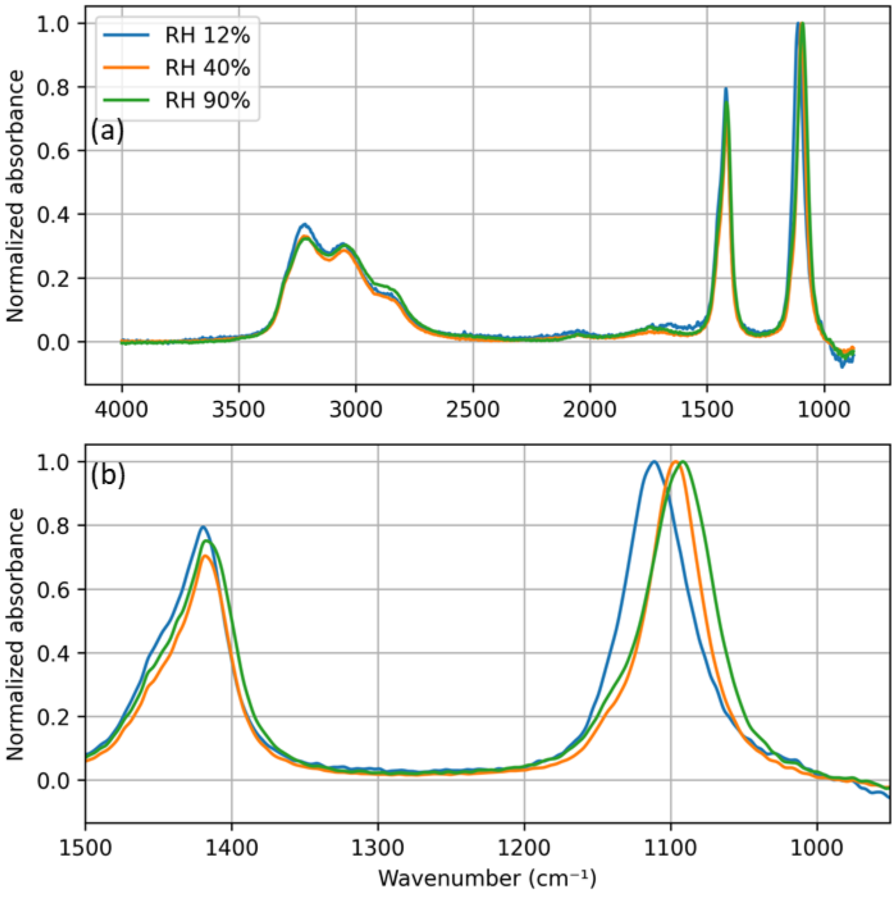


**Figure S3. NEMS-FTIR spectra of ammonium sulfate at different relative humidity.** Panel (a) shows the full spectra and panel (b) zooms in on the region 1500-1100 $cm^{-1}$

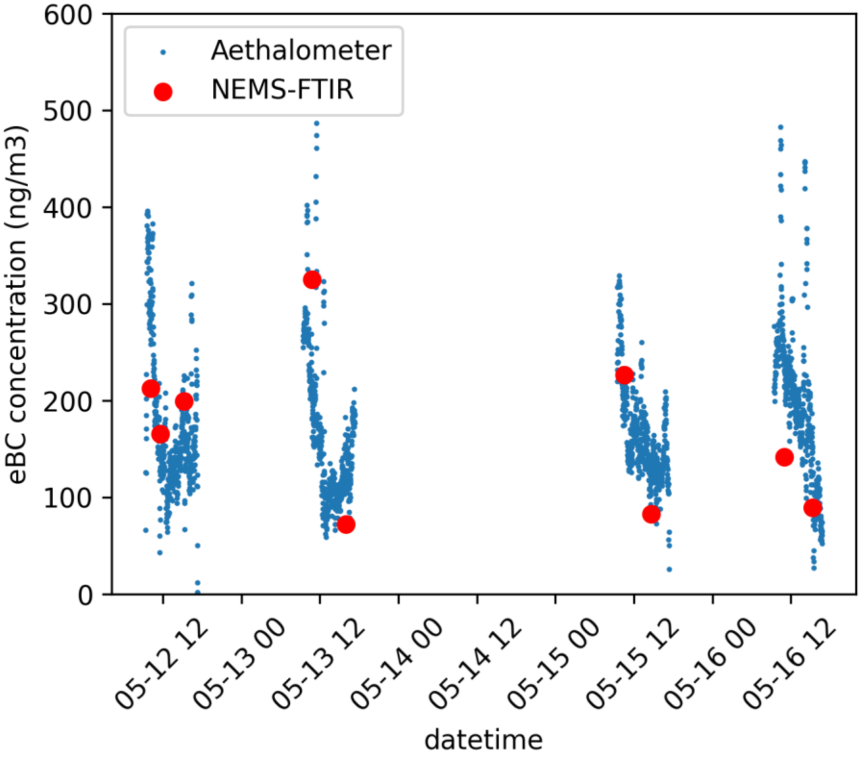


**Figure S4. Comparison of equivalent black carbon (eBC) from aethalometer and NEMS-FTIR over 4 different days.** Collection times for NEMS-FTIR range between 25-45 minutes.

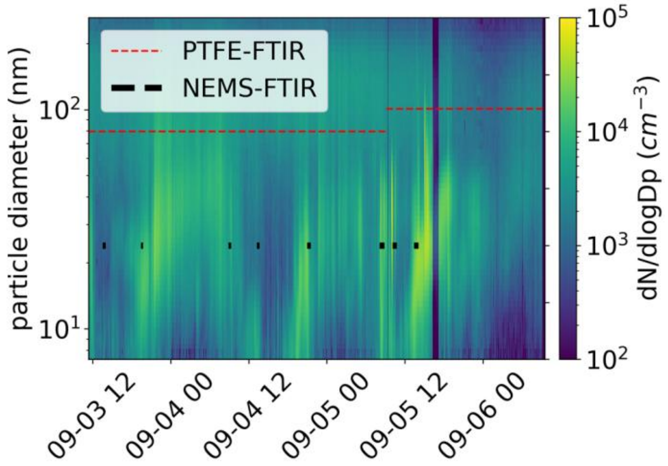


**Figure S5. Sampling times for collocated PTFE- and NEMS-FTIR measurements in Vienna.** The markers indicating the sampling duration are offset for clarity.

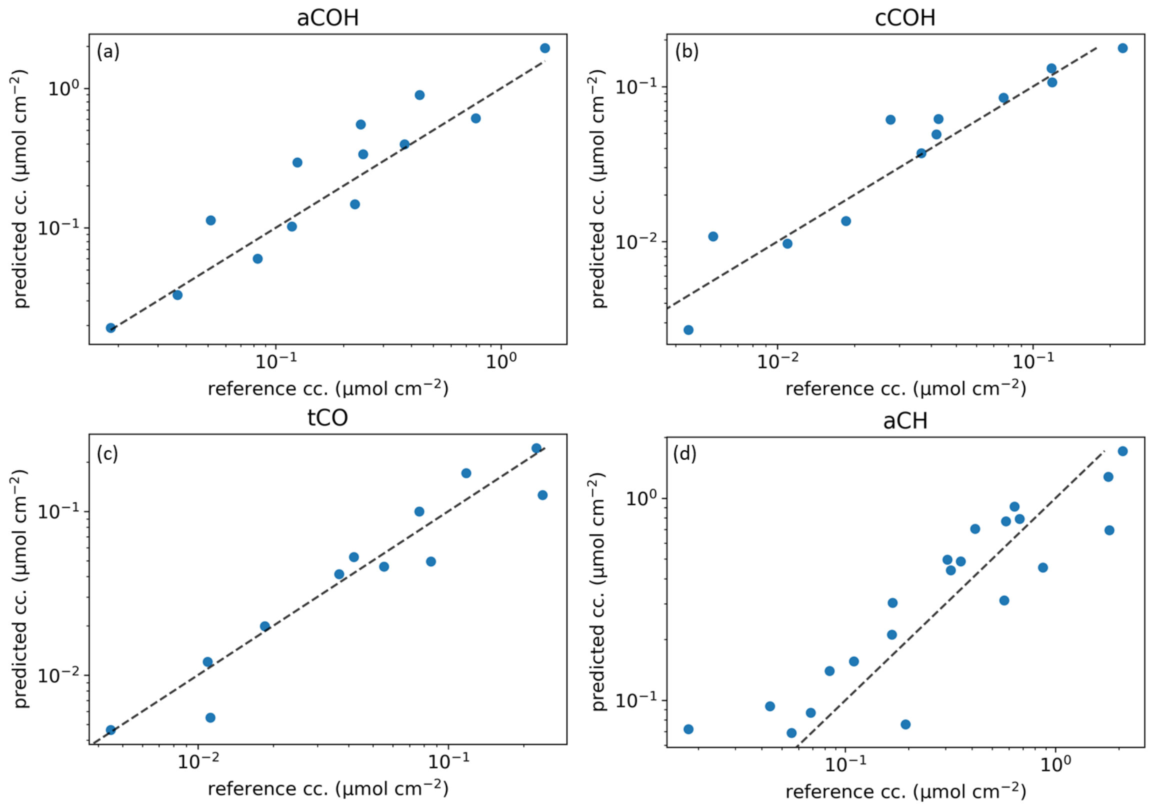


**Figure S6. Comparison of reference (from SMPS) and predicted (from NEMS-FTIR) concentrations for aCOH, cCOH, tCO and aCH in panels (a) through (d), respectively.** Dashed lines are 1:1 lines. The mean error is 20, 9.5, 13 and 31% for aCOH, cCOH, tCO and aCH, respectively.

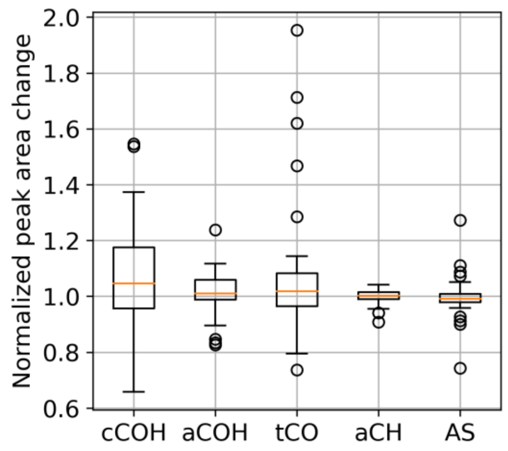


**Figure S7: Sensitivity test for baseline subtraction.** The smoothing parameter lambda was increased and decreased, respectively, by an order of magnitude compared to the initial "best match" chosen by visual inspection, for the ambient spectra (n=32).The peak area normalized to the "best match" peak area for the different functional groups and ammonium sulfate is shown. Orange line represents the mean, while box and whiskers show 25-75 and 10-90th percentiles, respectively.

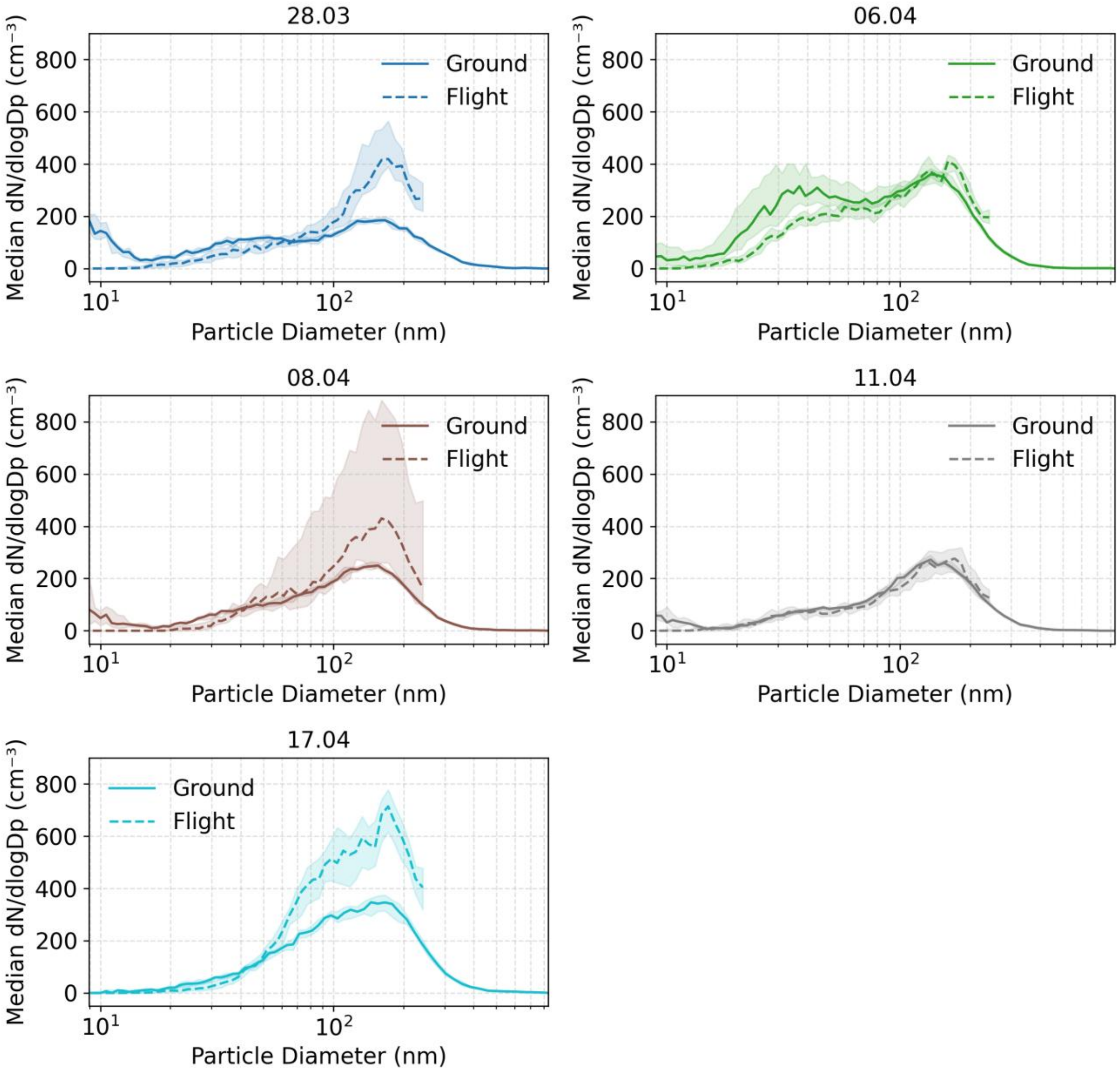


**Figure S8: Particle size distributions at the ground and aloft (altitude>200m) for sampling times of the NEMS resonators at Villum Research Station.** Lines represent the median and shaded areas are 25-75$^{th}$ percentile ranges. The size distributions are for different particle size ranges due to the different instruments used at the ground (mobility particle size spectrometer with size range 8-800 nm) and aloft (mSEMS with size range 2-240 nm).

## Text S2.Air mass trajectory modelling and emission sources.

The trajectories of airmasses arriving at Villum Research Station were examined using the LAGRANTO model supplied by 6-hourly ERA5 meteorological reanalysis *(90)*. Trajectories were set off every six hours from a group of starting positions arranged around the Villum Research Station. These start points are an evenly spaced grid of 25 km, created through the region.eqd(1,25) command in LAGRANTO. Back trajectories covering seven days were produced for pressures ranging from 1020 down to 900 hPa.

The ECLIPSE v6b emission inventory was used to provide estimates of emission sources of BC, CO and $SO_2$ *(91)* for the year 2020. Grid points with very low emission values below 0.001, 0.1 and 0.1 kt/year, for each pollutant, respectively were set to zero. Afterwards, the median for all

grid points was calculated. Grid points with values higher than the median were selected and are considered emission grid points. In addition, emissions from gas flaring were considered. Data from the Visible Infrared Imaging Radiometer Suite (VIIRS) satellite instrument taken from https://flaring.skytruth.org/ were used for gas flaring *(92)*.

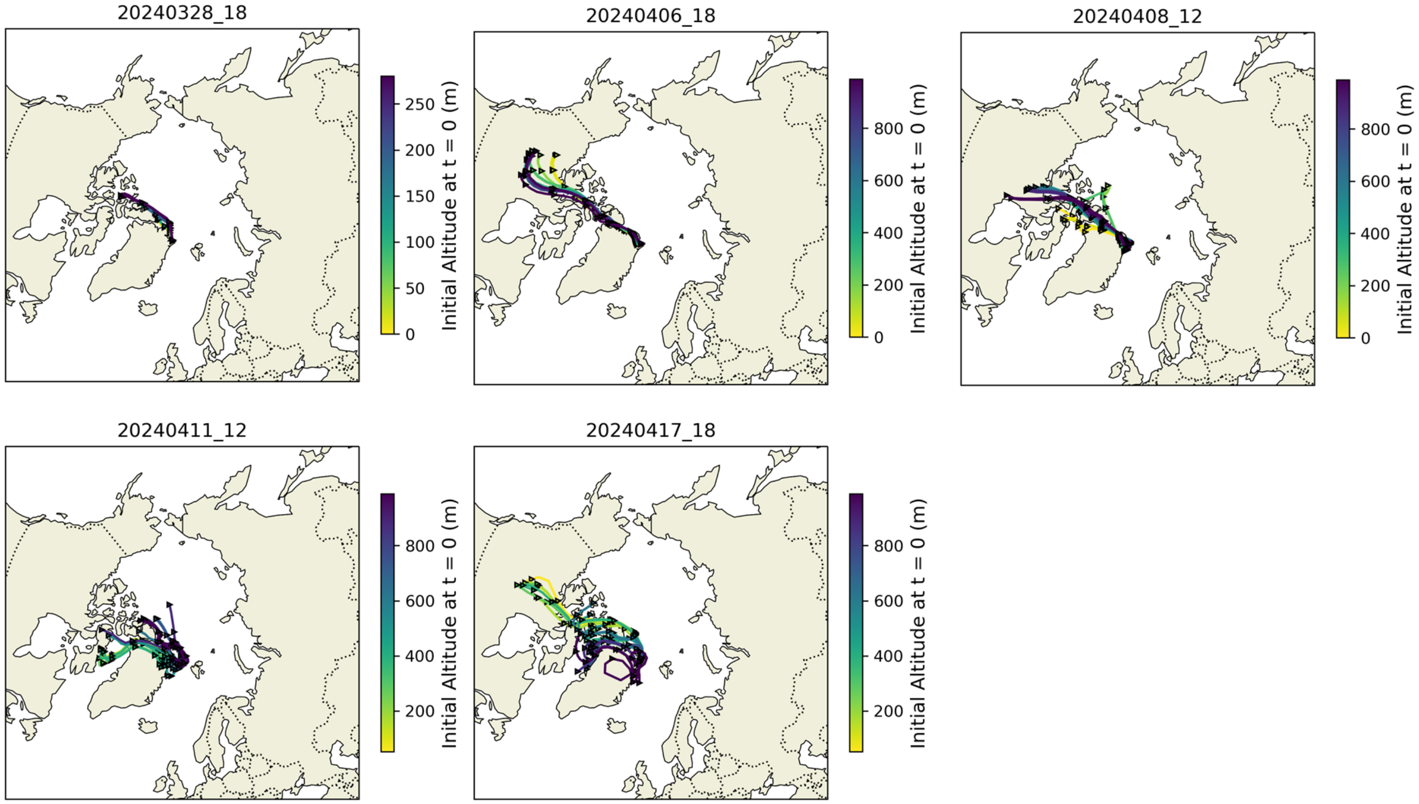


**Figure S9. Air mass back trajectories arriving at Villum Research Station, dated YYYYMMDD_HH.** All back trajectories are 7 days long, except that on the 28th of March which has been cut to 3 days due to degenerate trajectories (looping around the north pole). Markers represent 1 day of travel. Trajectories are colored by their release altitude. Trajectories were initialized using the 6-hour time step nearest to the NEMS sampling time.

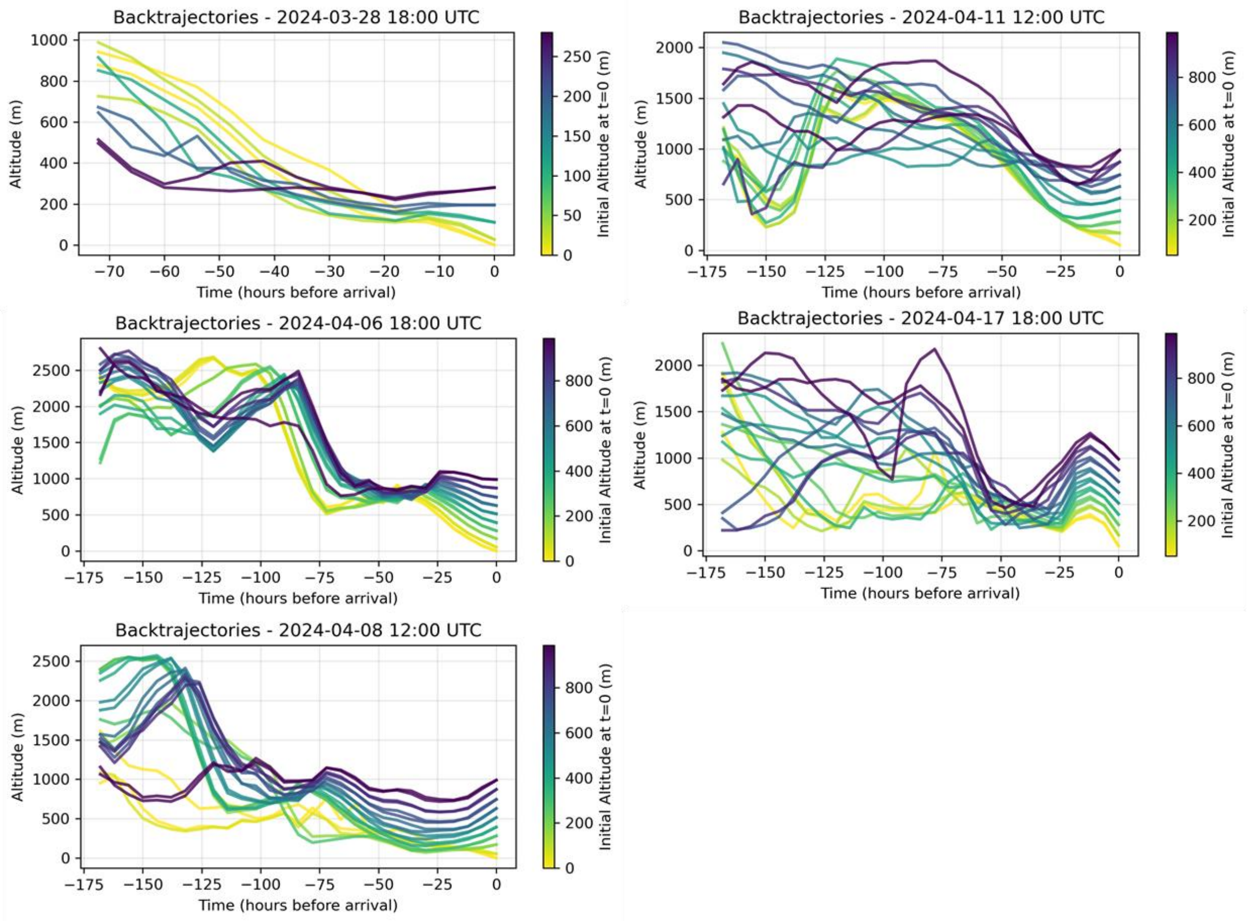


**Figure S10.** Altitude profiles of the back trajectories in Figure S9.

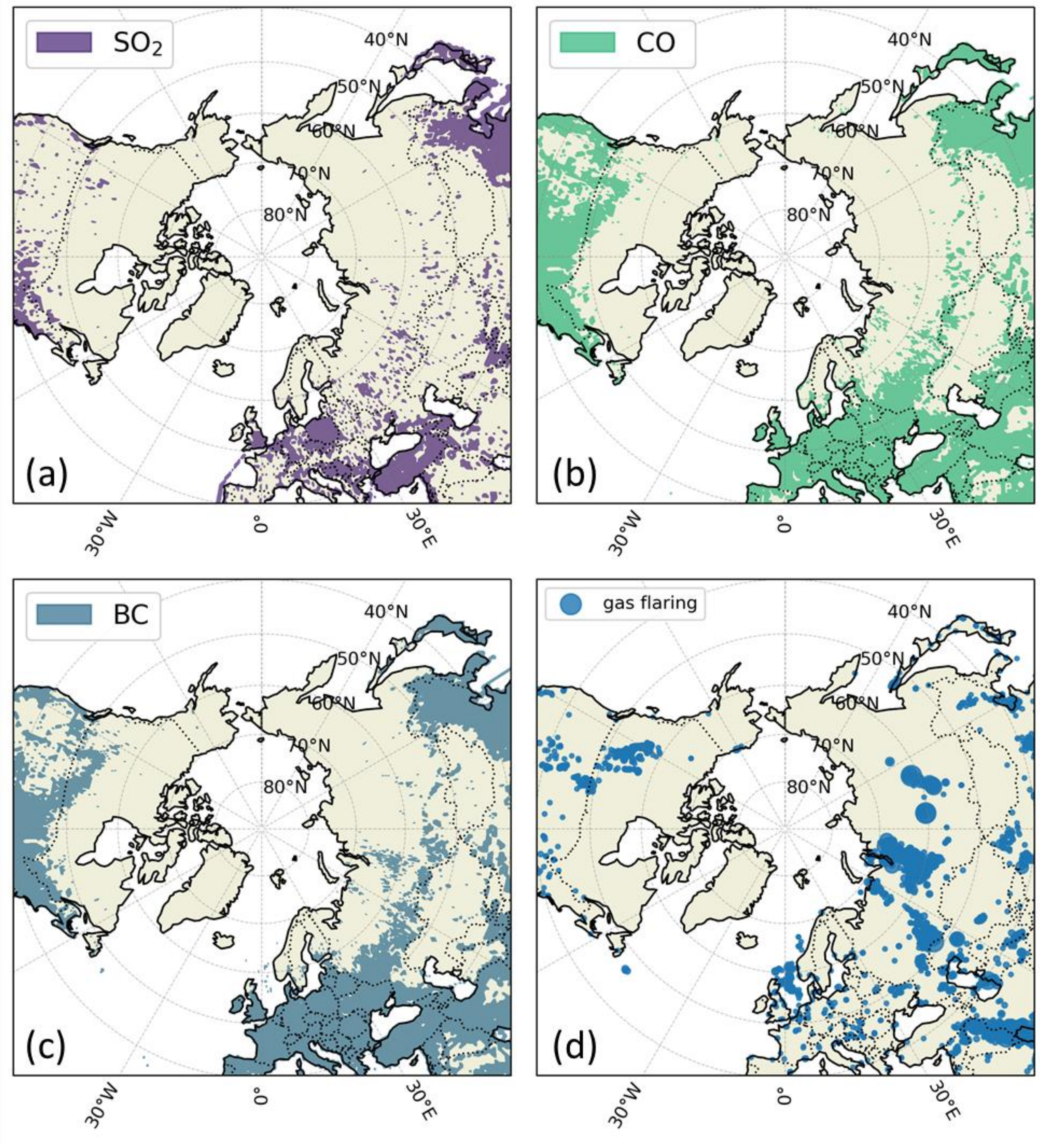


**Figure S11.** Emission maps for $SO_2$, CO and BC for 2020 in panels (a,b,c), respectively. Panel (d) shows identified gas flaring locations in 2020 sized by the volume of gas flared.

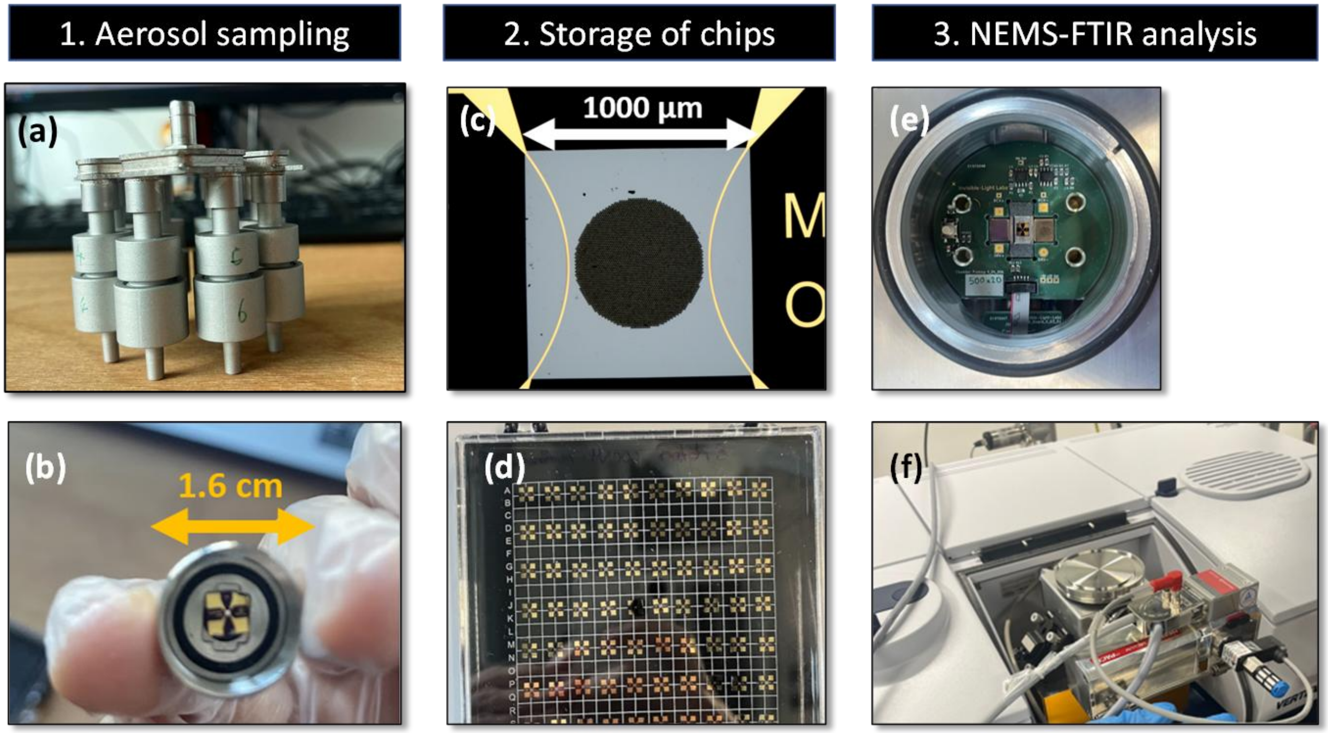


**Figure S12. Schematic overview of the NEMS-FTIR workflow, from sampling to analysis.** Panel (a) shows a modified stainless steel NEMS resonator holder (Aerosol Flow Adapter, Invisible-Light Labs GmbH, Austria) made for the multichannel sampler (FILT, Brechtel Manufacturing, Inc., USA). Panel (b) shows a NEMS resonator seated in the holder measuring 1.6 cm in diameter. Panel (c) shows an optical microscope close-up of the membrane of the NEMS resonator, heavily and homogenously sampled with aerosols in the perforated area. Panel (d) shows a sticky-gel carrier box containing the resonators for storage. The resonators are then brought back to the lab and analysed in the EMILIE analysis chamber (panel (e), Invisible-Light Labs GmbH, Austria) coupled to a commercial FTIR instrument, here the Bruker Vertex 70 (panel (f), Bruker Optics, Germany)

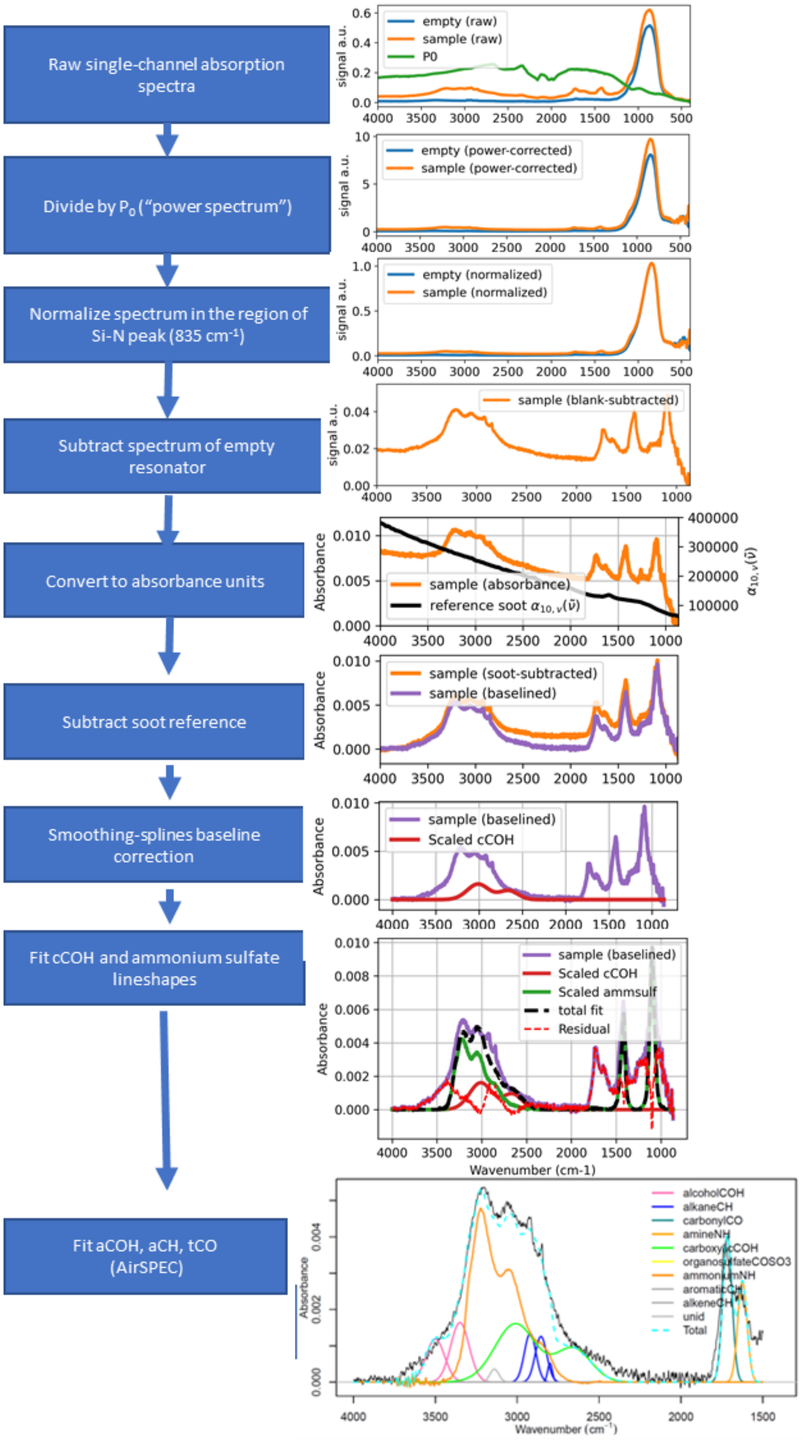


**Figure S13. Workflow and visualization of the spectral processing and peak-fitting steps for NEMS-FTIR.**

**Text S3. Derivation of sparse particle film and effective media approximation decadic linear attenuation coefficients**

If the particles are sparsely distributed and not interacting optically, they can be treated as individual non-interacting absorbers. For small particles, with a radius a lot smaller than the wavelength of the probing light ($R \ll \lambda$), the absorption cross section is given by *(93)*

$$\sigma_{abs}(\tilde{v}) = 8\,\pi^2 \tilde{v}\, R^3\, Im\left\{\frac{\tilde{n}^2(\tilde{v}) - \tilde{n}_m^2(\tilde{v})}{\tilde{n}^2(\tilde{v}) + 2\tilde{n}_m^2(\tilde{v})}\right\}, \quad \text{S1}$$

with the wavenumber $\tilde{v} = 1/\lambda$ and the complex refractive index of the particles $\tilde{n}(\tilde{v})$, and the complex refractive index of the surrounding medium $\tilde{n}_m(\tilde{v})$. The decadic volume attenuation coefficient of a particle is now given by

$$\alpha_{10,v}(\tilde{v}) = \frac{\sigma_{abs}(\tilde{v})}{\ln{(10)}}, \quad \text{S2}$$

which can be converted to the decadic linear attenuation coefficient by dividing by the particle volume

$$\alpha_{10}(\tilde{v}) = \frac{\alpha_{10,v}(\tilde{v})}{\frac{4}{3}\pi R^3} = \frac{6\pi\tilde{v}}{\ln{(10)}} Im\left\{\frac{\tilde{n}^2(\tilde{v}) - \tilde{n}_m^2(\tilde{v})}{\tilde{n}^2(\tilde{v}) + 2\tilde{n}_m^2(\tilde{v})}\right\}. \quad \text{S3}$$

The collected nanoparticles are positioned on top of a 50 nm-thick suspended silicon nitride resonator. Due to its subwavelength thickness, the silicon nitride layer is optically thin and does not significantly affect the effective refractive index of the surrounding medium, as confirmed by finite element simulations (Figure S7). Therefore, the refractive index of the surrounding medium can be approximated by that of vacuum, i.e., $\tilde{n}_m = 1$. The formulation in equation S3 for the decadic linear attenuation coefficient is applicable for particles smaller than the wavelength of the probing light, where absorption is the dominant contributor to attenuation and scattering is negligible.

As an alternative to the sparse particle film (SPF) model equation S3, the NEMS-FTIR spectra are also compared to an effective media approximation (EMA), assuming that the sample is a homogeneous film. In that case, the decadic linear attenuation coefficient is given by

$$\alpha_{10}^{(EMA)}(\tilde{v}) = \frac{4\pi\tilde{v}}{\ln{(10)}} Im\{\tilde{n}(\tilde{v})\}. \quad S4$$

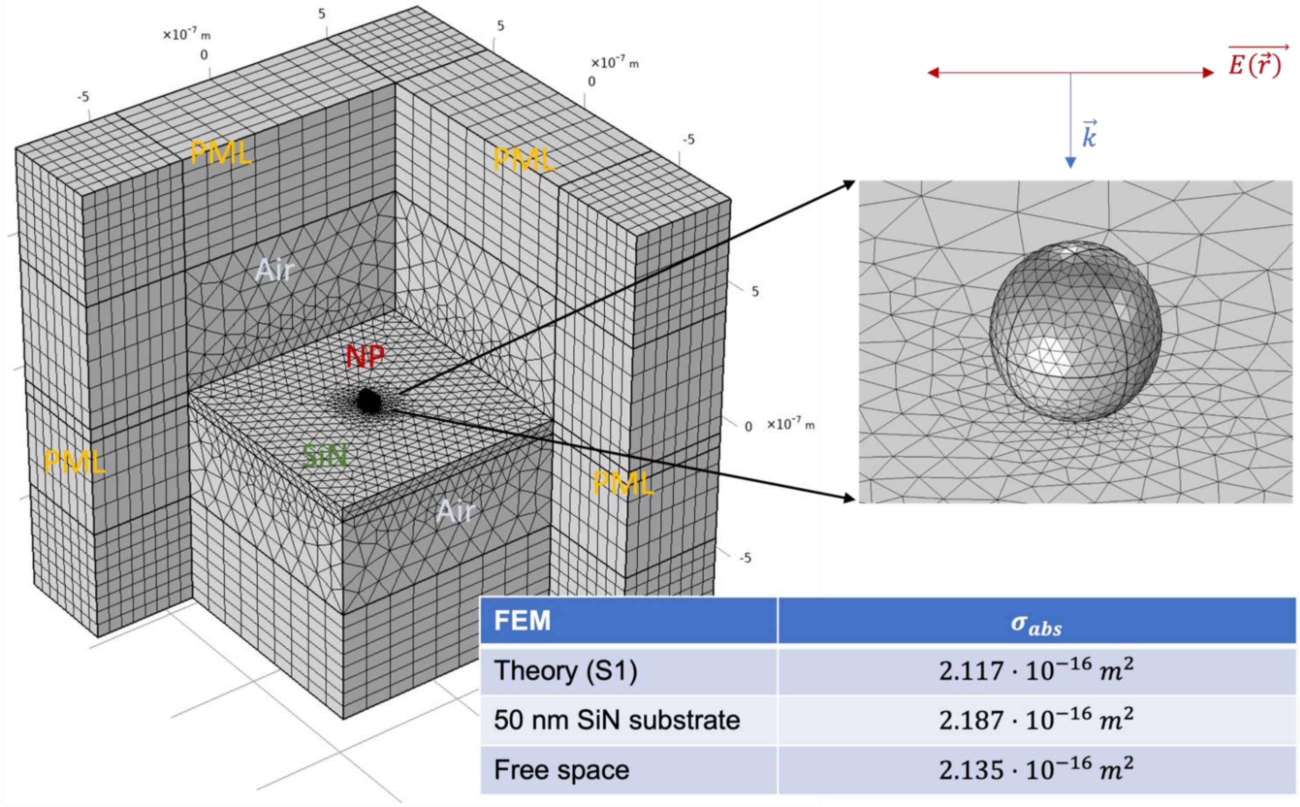


| FEM | $\sigma_{abs}$ |
|---|---|
| Theory (S1) | $2.117 \cdot 10^{-16}\ m^2$ |
| 50 nm SiN substrate | $2.187 \cdot 10^{-16}\ m^2$ |
| Free space | $2.135 \cdot 10^{-16}\ m^2$ |

**Figure S14. Finite element simulation of absorption cross section.** Model of a 100 nm ammonium sulfate [$\boldsymbol{n_{AS}(\lambda) = 2.85}$ and $\boldsymbol{\kappa_{AS}(\lambda) = 2}$] *(46)* nanoparticle on top of a 50 nm-thick silicon nitride film [$\boldsymbol{n_{SiN}(\lambda) = 1.5594}$ and $\boldsymbol{\kappa_{SiN}}(\boldsymbol{\lambda})$ =0.72509] (Obtained from FTIR transmission measurement of a 50nm thick silicon nitride membrane), probed at a wavenumber of 1100 cm$^{-1}$. Abbreviations PML and NP in the Figure refer to perfectly matched layer and nanoparticle, respectively.

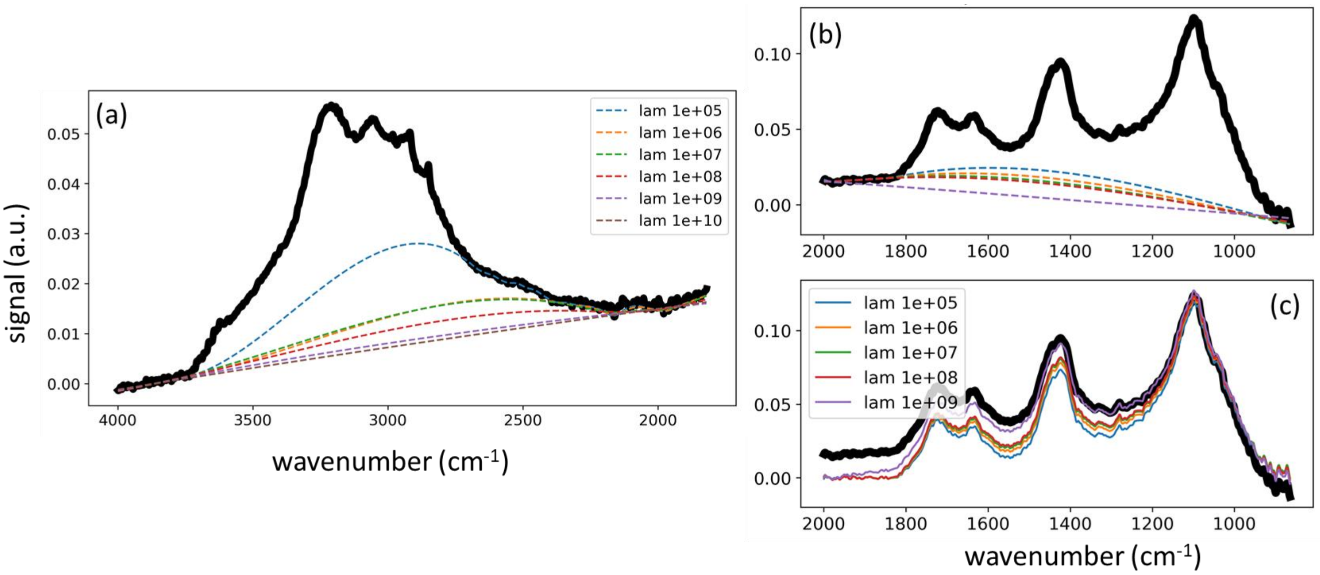


**Figure S15. Examples of baseline correction after soot subtraction.** Panels (a) as well as (b) and (c) show the two regions of the spectrum which were baseline-corrected separately, respectively. The dashed lines in (a) and (b) denote the baselines for each smoothing parameter lambda, the black solid line is the input spectrum and the solid-coloured lines in panel (c) are the baseline-corrected spectra for each smoothing parameter lambda. For this spectrum, a lambda of $1e^9$ and $1e^8$ was chosen for each spectral region, respectively.

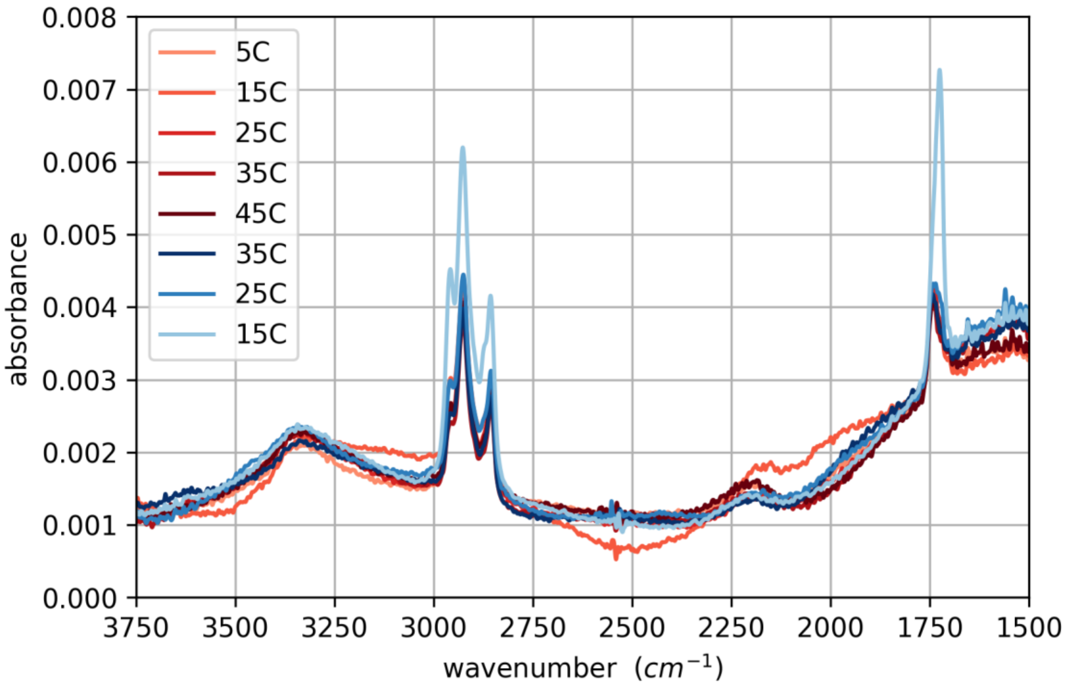


**Figure S16: Comparison of blank NEMS-FTIR spectra analyzed at different temperatures.** Red spectra were obtained upon sequential heating and blue spectra upon cooling, immediately thereafter.

## REFERENCES


1. Intergovernmental Panel on Climate Change (IPCC), *Climate Change 2021 – The Physical Science Basis: Working Group I Contribution to the Sixth Assessment Report of the Intergovernmental Panel on Climate Change* (Cambridge Univ. Press, 2023); www.cambridge.org/core/books/climate-change-2021-the-physical-science-basis/415F29233B8BD19FB55F65E3DC67272B.

2. K. R. Daellenbach, G. Uzu, J. Jiang, L.-E. Cassagnes, Z. Leni, A. Vlachou, G. Stefenelli, F. Canonaco, S. Weber, A. Segers, J. J. P. Kuenen, M. Schaap, O. Favez, A. Albinet, S. Aksoyoglu, J. Dommen, U. Baltensperger, M. Geiser, I. El Haddad, J.-L. Jaffrezo, A. S. H. Prévôt, Sources of particulate-matter air pollution and its oxidative potential in Europe. *Nature* **587**, 414–419 (2020).

3. J. Lelieveld, J. S. Evans, M. Fnais, D. Giannadaki, A. Pozzer, The contribution of outdoor air pollution sources to premature mortality on a global scale. *Nature* **525**, 367–371 (2015).

4. J. Merikanto, D. V. Spracklen, G. W. Mann, S. J. Pickering, K. S. Carslaw, Impact of nucleation on global CCN. *Atmos. Chem. Phys.* **9**, 8601–8616 (2009).

5. H. Gordon, J. Kirkby, U. Baltensperger, F. Bianchi, M. Breitenlechner, J. Curtius, A. Dias, J. Dommen, N. M. Donahue, E. M. Dunne, J. Duplissy, S. Ehrhart, R. C. Flagan, C. Frege, C. Fuchs, A. Hansel, C. R. Hoyle, M. Kulmala, A. Kürten, K. Lehtipalo, V. Makhmutov, U. Molteni, M. P. Rissanen, Y. Stozkhov, J. Tröstl, G. Tsagkogeorgas, R. Wagner, C. Williamson, D. Wimmer, P. M. Winkler, C. Yan, K. S. Carslaw, Causes and importance of new particle formation in the present-day and preindustrial atmospheres. *J. Geophys. Res. Atmos.* **122**, 8739–8760 (2017).

6. S.-H. Lee, H. Gordon, H. Yu, K. Lehtipalo, R. Haley, Y. Li, R. Zhang, New particle formation in the atmosphere: From molecular clusters to global climate. *J. Geophys. Res. Atmos.* **124**, 7098–7146 (2019).

7. R. A. Zaveri, J. Wang, J. Fan, Y. Zhang, J. E. Shilling, A. Zelenyuk, F. Mei, R. Newsom, M. Pekour, J. Tomlinson, J. M. Comstock, M. Shrivastava, E. Fortner, L. A. T. Machado, P.

Artaxo, S. T. Martin, Rapid growth of anthropogenic organic nanoparticles greatly alters cloud life cycle in the Amazon rainforest. *Sci. Adv.* **8**, eabj0329 (2022).

8. A. Peters, H. E. Wichmann, T. Tuch, J. Heinrich, J. Heyder, Respiratory effects are associated with the number of ultrafine particles. *Am. J. Respir. Crit. Care Med.* **155**, 1376–1383 (1997).

9. D. E. Schraufnagel, The health effects of ultrafine particles. *Exp. Mol. Med.* **52**, 311–317 (2020).

10. R. Pohorsky, A. Baccarini, N. Brett, B. Barret, S. Bekki, G. Pappaccogli, E. Dieudonné, B. Temime-Roussel, B. D'Anna, M. Cesler-Maloney, A. Donateo, S. Decesari, K. S. Law, W. R. Simpson, J. Fochesatto, S. R. Arnold, J. Schmale, In situ vertical observations of the layered structure of air pollution in a continental high-latitude urban boundary layer during winter. *Atmos. Chem. Phys.* **25**, 3687–3715 (2025).

11. M. R. Canagaratna, J. T. Jayne, J. L. Jimenez, J. D. Allan, M. R. Alfarra, Q. Zhang, T. B. Onasch, F. Drewnick, H. Coe, A. Middlebrook, A. Delia, L. R. Williams, A. M. Trimborn, M. J. Northway, P. F. DeCarlo, C. E. Kolb, P. Davidovits, D. R. Worsnop, Chemical and microphysical characterization of ambient aerosols with the aerodyne aerosol mass spectrometer. *Mass Spectrom. Rev.* **26**, 185–222 (2007).

12. P. F. DeCarlo, J. R. Kimmel, A. Trimborn, M. J. Northway, J. T. Jayne, A. C. Aiken, M. Gonin, K. Fuhrer, T. Horvath, K. S. Docherty, D. R. Worsnop, J. L. Jimenez, Field-deployable, high-resolution, time-of-flight aerosol mass spectrometer. *Anal. Chem.* **78**, 8281–8289 (2006).

13. J. T. Jayne, D. C. Leard, X. Zhang, P. Davidovits, K. A. Smith, C. E. Kolb, D. R. Worsnop, Development of an aerosol mass spectrometer for size and composition analysis of submicron particles. *Aerosol Sci. Tech.* **33**, 49–70 (2000).

14. Q. Zhang, J. L. Jimenez, M. R. Canagaratna, I. M. Ulbrich, N. L. Ng, D. R. Worsnop, Y. Sun, Understanding atmospheric organic aerosols via factor analysis of aerosol mass spectrometry: A review. *Anal. Bioanal. Chem.* **401**, 3045–3067 (2011).

15. J. L. Jimenez, M. R. Canagaratna, N. M. Donahue, A. S. H. Prevot, Q. Zhang, J. H. Kroll, P. F. DeCarlo, J. D. Allan, H. Coe, N. L. Ng, A. C. Aiken, K. S. Docherty, I. M. Ulbrich, A. P. Grieshop, A. L. Robinson, J. Duplissy, J. D. Smith, K. R. Wilson, V. A. Lanz, C. Hueglin, Y. L. Sun, J. Tian, A. Laaksonen, T. Raatikainen, J. Rautiainen, P. Vaattovaara, M. Ehn, M. Kulmala, J. M. Tomlinson, D. R. Collins, M. J. Cubison, E. J. Dunlea, J. A. Huffman, T. B. Onasch, M. R. Alfarra, P. I. Williams, K. Bower, Y. Kondo, J. Schneider, F. Drewnick, S. Borrmann, S. Weimer, K. Demerjian, D. Salcedo, L. Cottrell, R. Griffin, A. Takami, T. Miyoshi, S. Hatakeyama, A. Shimono, J. Y. Sun, Y. M. Zhang, K. Dzepina, J. R. Kimmel, D. Sueper, J. T. Jayne, S. C. Herndon, A. M. Trimborn, L. R. Williams, E. C. Wood, A. M. Middlebrook, C. E. Kolb, U. Baltensperger, D. R. Worsnop, Evolution of organic aerosols in the atmosphere. *Science* **326**, 1525–1529 (2009).

16. M. Crippa, F. Canonaco, V. A. Lanz, M. Äijälä, J. D. Allan, S. Carbone, G. Capes, D. Ceburnis, M. Dall'Osto, D. A. Day, P. F. DeCarlo, M. Ehn, A. Eriksson, E. Freney, L. Hildebrandt Ruiz, R. Hillamo, J. L. Jimenez, H. Junninen, A. Kiendler-Scharr, A.-M. Kortelainen, M. Kulmala, A. Laaksonen, A. A. Mensah, C. Mohr, E. Nemitz, C. O'Dowd, J. Ovadnevaite, S. N. Pandis, T. Petäjä, L. Poulain, S. Saarikoski, K. Sellegri, E. Swietlicki, P. Tiitta, D. R. Worsnop, U. Baltensperger, A. S. H. Prévôt, Organic aerosol components derived from 25 AMS data sets across Europe using a consistent ME-2 based source apportionment approach. *Atmos. Chem. Phys.* **14**, 6159–6176 (2014).

17. P. S. K. Liu, R. Deng, K. A. Smith, L. R. Williams, J. T. Jayne, M. R. Canagaratna, K. Moore, T. B. Onasch, D. R. Worsnop, T. Deshler, Transmission efficiency of an aerodynamic focusing lens system: Comparison of model calculations and laboratory measurements for the aerodyne aerosol mass spectrometer. *Aerosol Sci. Tech.* **41**, 721–733 (2007).

18. X. Zhang, K. A. Smith, D. R. Worsnop, J. Jimenez, J. T. Jayne, C. E. Kolb, A numerical characterization of particle beam collimation by an aerodynamic lens-nozzle system: Part I. An individual lens or nozzle. *Aerosol Sci. Tech.* **36**, 617–631 (2002).

19. F. D. Lopez-Hilfiker, C. Mohr, M. Ehn, F. Rubach, E. Kleist, J. Wildt, T. F. Mentel, A. Lutz, M. Hallquist, D. Worsnop, J. A. Thornton, A novel method for online analysis of gas and

particle composition: Description and evaluation of a Filter Inlet for Gases and AEROsols (FIGAERO). *Atmos. Meas. Tech.* **7**, 983–1001 (2014).

20. F. D. Lopez-Hilfiker, V. Pospisilova, W. Huang, M. Kalberer, C. Mohr, G. Stefenelli, J. A. Thornton, U. Baltensperger, A. S. H. Prevot, J. G. Slowik, An extractive electrospray ionization time-of-flight mass spectrometer (EESI-TOF) for online measurement of atmospheric aerosol particles. *Atmos. Meas. Tech.* **12**, 4867–4886 (2019).

21. R. Holzinger, J. Williams, F. Herrmann, J. Lelieveld, N. M. Donahue, T. Röckmann, Aerosol analysis using a Thermal-Desorption Proton-Transfer-Reaction Mass Spectrometer (TD-PTR-MS): A new approach to study processing of organic aerosols. *Atmos. Chem. Phys.* **10**, 2257–2267 (2010).

22. T. Nah, M. Chan, S. R. Leone, K. R. Wilson, Real time in situ chemical characterization of submicrometer organic particles using direct analysis in real time-mass spectrometry. *Anal. Chem.* **85**, 2087–2095 (2013).

23. M. Müller, P. Eichler, B. D'Anna, W. Tan, A. Wisthaler, Direct sampling and analysis of atmospheric particulate organic matter by proton-transfer-reaction mass spectrometry. *Anal. Chem.* **89**, 10889–10897 (2017).

24. Q. Ye, M. Wang, V. Hofbauer, D. Stolzenburg, D. Chen, M. Schervish, A. Vogel, R. L. Mauldin, R. Baalbaki, S. Brilke, L. Dada, A. Dias, J. Duplissy, I. El Haddad, H. Finkenzeller, L. Fischer, X. He, C. Kim, A. Kürten, H. Lamkaddam, C. P. Lee, K. Lehtipalo, M. Leiminger, H. E. Manninen, R. Marten, B. Mentler, E. Partoll, T. Petäjä, M. Rissanen, S. Schobesberger, S. Schuchmann, M. Simon, Y. J. Tham, M. Vazquez-Pufleau, A. C. Wagner, Y. Wang, Y. Wu, M. Xiao, U. Baltensperger, J. Curtius, R. Flagan, J. Kirkby, M. Kulmala, R. Volkamer, P. M. Winkler, D. Worsnop, N. M. Donahue, Molecular composition and volatility of nucleated particles from α-pinene oxidation between −50°C and +25°C. *Environ. Sci. Technol.* **53**, 12357–12365 (2019).

25. M. Surdu, V. Pospisilova, M. Xiao, M. Wang, B. Mentler, M. Simon, D. Stolzenburg, C. R. Hoyle, D. M. Bell, C. Ping Lee, H. Lamkaddam, F. Lopez-Hilfiker, L. R. Ahonen, A. Amorim, A. Baccarini, D. Chen, L. Dada, J. Duplissy, H. Finkenzeller, X.-C. He, V.

Hofbauer, C. Kim, A. Kürten, A. Kvashnin, K. Lehtipalo, V. Makhmutov, U. Molteni, W. Nie, A. Onnela, T. Petäjä, L. L. J. Quéléver, C. Tauber, A. Tomé, R. Wagner, C. Yan, A. S. H. Prevot, J. Dommen, N. M. Donahue, A. Hansel, J. Curtius, P. M. Winkler, M. Kulmala, R. Volkamer, R. C. Flagan, J. Kirkby, D. R. Worsnop, J. G. Slowik, D. S. Wang, U. Baltensperger, I. el Haddad, Molecular characterization of ultrafine particles using extractive electrospray time-of-flight mass spectrometry. *Environ. Sci. Atmos.* **1**, 434–448 (2021).

26. X. Li, Y. Li, M. J. Lawler, J. Hao, J. N. Smith, J. Jiang, Composition of ultrafine particles in urban Beijing: Measurement using a thermal desorption chemical ionization mass spectrometer. *Environ. Sci. Technol.* **55**, 2859–2868 (2021).

27. X. Li, Y. Li, R. Cai, C. Yan, X. Qiao, Y. Guo, C. Deng, R. Yin, Y. Chen, Y. Li, L. Yao, N. Sarnela, Y. Zhang, T. Petäjä, F. Bianchi, Y. Liu, M. Kulmala, J. Hao, J. N. Smith, J. Jiang, Insufficient condensable organic vapors lead to slow growth of new particles in an urban environment. *Environ. Sci. Technol.* **56**, 9936–9946 (2022).

28. J. Liu, J. Dedrick, L. M. Russell, G. I. Senum, J. Uin, C. Kuang, S. R. Springston, W. R. Leaitch, A. C. Aiken, D. Lubin, High summertime aerosol organic functional group concentrations from marine and seabird sources at Ross Island, Antarctica, during AWARE. *Atmos. Chem. Phys.* **18**, 8571–8587 (2018).

29. S. F. Maria, L. M. Russell, B. J. Turpin, R. J. Porcja, T. L. Campos, R. J. Weber, B. J. Huebert, Source signatures of carbon monoxide and organic functional groups in Asian Pacific Regional Aerosol Characterization Experiment (ACE-Asia) submicron aerosol types. *J. Geophys. Res. Atmos.* **108**, 8637 (2003).

30. L. M. Russell, R. Bahadur, P. J. Ziemann, Identifying organic aerosol sources by comparing functional group composition in chamber and atmospheric particles. *Proc. Natl. Acad. Sci. U.S.A.* **108**, 3516–3521 (2011).

31. S. Takahama, R. E. Schwartz, L. M. Russell, A. M. Macdonald, S. Sharma, W. R. Leaitch, Organic functional groups in aerosol particles from burning and non-burning forest emissions at a high-elevation mountain site. *Atmos. Chem. Phys.* **11**, 6367–6386 (2011).

32. M. S. Claflin, J. E. Krechmer, W. Hu, J. L. Jimenez, P. J. Ziemann, Functional group composition of secondary organic aerosol formed from ozonolysis of α-pinene under high VOC and autoxidation conditions. *ACS Earth Space Chem.* **2**, 1196–1210 (2018).

33. J. F. Pankow, W. E. Asher, SIMPOL.1: A simple group contribution method for predicting vapor pressures and enthalpies of vaporization of multifunctional organic compounds. *Atmos. Chem. Phys.* **8**, 2773–2796 (2008).

34. M. Shiraiwa, K. Ueda, A. Pozzer, G. Lammel, C. J. Kampf, A. Fushimi, S. Enami, A. M. Arangio, J. Fröhlich-Nowoisky, Y. Fujitani, A. Furuyama, P. S. J. Lakey, J. Lelieveld, K. Lucas, Y. Morino, U. Pöschl, S. Takahama, A. Takami, H. Tong, B. Weber, A. Yoshino, K. Sato, Aerosol health effects from molecular to global scales. *Environ. Sci. Technol.* **51**, 13545–13567 (2017).

35. N. E. Rothfuss, M. D. Petters, Influence of functional groups on the viscosity of organic aerosol. *Environ. Sci. Technol.* **51**, 271–279 (2017).

36. S. R. Suda, M. D. Petters, G. K. Yeh, C. Strollo, A. Matsunaga, A. Faulhaber, P. J. Ziemann, A. J. Prenni, C. M. Carrico, R. C. Sullivan, S. M. Kreidenweis, Influence of functional groups on organic aerosol cloud condensation nucleus activity. *Environ. Sci. Technol.* **48**, 10182–10190 (2014).

37. B. Heutte, N. Bergner, H. Angot, J. B. Pernov, L. Dada, J. A. Mirrielees, I. Beck, A. Baccarini, M. Boyer, J. M. Creamean, K. R. Daellenbach, I. El Haddad, M. M. Frey, S. Henning, T. Laurila, V. Moschos, T. Petäjä, K. A. Pratt, L. L. J. Quéléver, M. D. Shupe, P. Zieger, T. Jokinen, J. Schmale, Observations of high-time-resolution and size-resolved aerosol chemical composition and microphysics in the central Arctic: Implications for climate-relevant particle properties. *Atmos. Chem. Phys.* **25**, 2207–2241 (2025).

38. P. Q. Fu, K. Kawamura, J. Chen, B. Charrière, R. Sempéré, Organic molecular composition of marine aerosols over the Arctic Ocean in summer: Contributions of primary emission and secondary aerosol formation. *Biogeosciences* **10**, 653–667 (2013).

39. J. Jang, K.-T. Park, Y. J. Yoon, S.-Y. Ha, E. Jang, K. H. Cho, J. Y. Lee, J. Park, Molecular-level chemical composition of aerosol and its potential source tracking at Antarctic Peninsula. *Environ. Res.* **239**, 117217 (2023).

40. A. J. Andersen, S. Yamada, E. K. Pramodkumar, T. L. Andresen, A. Boisen, S. Schmid, Nanomechanical IR spectroscopy for fast analysis of liquid-dispersed engineered nanomaterials. *Sens. Actuators B* **233**, 667–673 (2016).

41. T. S. Biswas, N. Miriyala, C. Doolin, X. Liu, T. Thundat, J. P. Davis, Femtogram-scale photothermal spectroscopy of explosive molecules on nanostrings. *Anal. Chem.* **86**, 11368–11372 (2014).

42. M. Kurek, M. Carnoy, P. E. Larsen, L. H. Nielsen, O. Hansen, T. Rades, S. Schmid, A. Boisen, Nanomechanical infrared spectroscopy with vibrating filters for pharmaceutical analysis. *Angew. Chem. Int. Ed. Engl.* **56**, 3901–3905 (2017).

43. N. Luhmann, R. G. West, J. P. Lafleur, S. Schmid, Nanoelectromechanical infrared spectroscopy with in situ separation by thermal desorption: NEMS-IR-TD. *ACS Sens.* **8**, 1462–1470 (2023).

44. F. Samaeifar, A. Casci Ceccacci, S. Bose Goswami, L. Hagner Nielsen, A. Afifi, K. Zór, A. Boisen, Evaluation of the solid state form of tadalafil in sub-micron thin films using nanomechanical infrared spectroscopy. *Int. J. Pharm.* **565**, 227–232 (2019).

45. S. Yamada, S. Schmid, T. Larsen, O. Hansen, A. Boisen, Photothermal infrared spectroscopy of airborne samples with mechanical string resonators. *Anal. Chem.* **85**, 10531–10535 (2013).

46. M. E. Earle, R. G. Pancescu, B. Cosic, A. Y. Zasetsky, J. J. Sloan, Temperature-dependent complex indices of refraction for crystalline $(NH_4)_2SO_4$. *J. Phys. Chem. A* **110**, 13022–13028 (2006).

47. N. Dudani, S. Takahama, Design and fabrication of an electrostatic precipitator for infrared spectroscopy. *Atmos. Meas. Tech.* **15**, 4693–4707 (2022).

48. O. Laskina, M. A. Young, P. D. Kleiber, V. H. Grassian, Infrared optical constants of organic aerosols: Organic Acids and Model Humic-Like Substances (HULIS). *Aerosol Sci. Tech.* **48**, 630–637 (2014).

49. T. L. Myers, T. A. Blake, M. O. Yokosuk, G. Fortin, T. J. Johnson, Improved infrared optical constants from pressed pellets: II. Ellipsometric n and k values for ammonium sulfate with variability analysis. *Appl. Spectrosc.* **74**, 868–882 (2020).

50. S. Takahama, A. Johnson, L. M. Russell, Quantification of carboxylic and carbonyl functional groups in organic aerosol infrared absorbance spectra. *Aerosol Sci. Tech.* **47**, 310–325 (2013).

51. M. Reggente, A. M. Dillner, S. Takahama, Analysis of functional groups in atmospheric aerosols by infrared spectroscopy: Systematic intercomparison of calibration methods for US measurement network samples. *Atmos. Meas. Tech.* **12**, 2287–2312 (2019).

52. J. Coates, "Interpretation of infrared spectra, a practical approach," in *Encyclopedia of Analytical Chemistry* (John Wiley & Sons, Ltd, 2000), pp. 10815–10837; https://onlinelibrary.wiley.com/doi/abs/10.1002/9780470027318.a5606.

53. A. J. Boris, S. Takahama, A. T. Weakley, B. M. Debus, C. D. Fredrickson, M. Esparza-Sanchez, C. Burki, M. Reggente, S. L. Shaw, E. S. Edgerton, A. M. Dillner, Quantifying organic matter and functional groups in particulate matter filter samples from the southeastern United States – Part 1: Methods. *Atmos. Meas. Tech.* **12**, 5391–5415 (2019).

54. V. Moschos, J. Schmale, W. Aas, S. Becagli, G. Calzolai, K. Eleftheriadis, C. E. Moffett, J. Schnelle-Kreis, M. Severi, S. Sharma, H. Skov, M. Vestenius, W. Zhang, H. Hakola, H. Hellén, L. Huang, J.-L. Jaffrezo, A. Massling, J. K. Nøjgaard, T. Petäjä, O. Popovicheva, R. J. Sheesley, R. Traversi, K. E. Yttri, A. S. H. Prévôt, U. Baltensperger, I. El Haddad, Elucidating the present-day chemical composition, seasonality and source regions of climate-relevant aerosols across the Arctic land surface. *Environ. Res. Lett.* **17**, 034032 (2022).

55. R. Bahreini, B. Ervens, A. M. Middlebrook, C. Warneke, J. A. de Gouw, P. F. DeCarlo, J. L. Jimenez, C. A. Brock, J. A. Neuman, T. B. Ryerson, H. Stark, E. Atlas, J. Brioude, A. Fried, J.

S. Holloway, J. Peischl, D. Richter, J. Walega, P. Weibring, A. G. Wollny, F. C. Fehsenfeld, Organic aerosol formation in urban and industrial plumes near Houston and Dallas, Texas. *J. Geophys. Res. Atmos.* **114**, D00F16 (2009).

56. A. Yazdani, N. Dudani, S. Takahama, A. Bertrand, A. S. H. Prévôt, I. El Haddad, A. M. Dillner, Fragment ion–functional group relationships in organic aerosols using aerosol mass spectrometry and mid-infrared spectroscopy. *Atmos. Meas. Tech.* **15**, 2857–2874 (2022).

57. S. Nakao, P. Tang, X. Tang, C. H. Clark, L. Qi, E. Seo, A. Asa-Awuku, D. Cocker, Density and elemental ratios of secondary organic aerosol: Application of a density prediction method. *Atmos. Environ.* **68**, 273–277 (2013).

58. M. Pitz, J. Cyrys, E. Karg, A. Wiedensohler, H.-E. Wichmann, J. Heinrich, Variability of apparent particle density of an urban aerosol. *Environ. Sci. Technol.* **37**, 4336–4342 (2003).

59. E. S. Cross, J. G. Slowik, P. Davidovits, J. D. Allan, D. R. Worsnop, J. T. Jayne, D. K. Lewis, M. Canagaratna, T. B. Onasch, Laboratory and ambient particle density determinations using light scattering in conjunction with aerosol mass spectrometry. *Aerosol Sci. Tech.* **41**, 343–359 (2007).

60. A. Khlystov, C. Stanier, S. N. Pandis, An algorithm for combining electrical mobility and aerodynamic size distributions data when measuring ambient aerosol. *Aerosol Sci. Tech.* **38**, 229–238 (2004).

61. S. Takahama, G. Ruggeri, Technical note: Relating functional group measurements to carbon types for improved model–measurement comparisons of organic aerosol composition. *Atmos. Chem. Phys.* **17**, 4433–4450 (2017).

62. D. Stolzenburg, L. Fischer, A. L. Vogel, M. Heinritzi, M. Schervish, M. Simon, A. C. Wagner, L. Dada, L. R. Ahonen, A. Amorim, A. Baccarini, P. S. Bauer, B. Baumgartner, A. Bergen, F. Bianchi, M. Breitenlechner, S. Brilke, S. Buenrostro Mazon, D. Chen, A. Dias, D. C. Draper, J. Duplissy, I. El Haddad, H. Finkenzeller, C. Frege, C. Fuchs, O. Garmash, H. Gordon, X. He, J. Helm, V. Hofbauer, C. R. Hoyle, C. Kim, J. Kirkby, J. Kontkanen, A. Kürten, J. Lampilahti, M. Lawler, K. Lehtipalo, M. Leiminger, H. Mai, S. Mathot, B. Mentler, U.

Molteni, W. Nie, T. Nieminen, J. B. Nowak, A. Ojdanic, A. Onnela, M. Passananti, T. Petäjä, L. L. J. Quéléver, M. P. Rissanen, N. Sarnela, S. Schallhart, C. Tauber, A. Tomé, R. Wagner, M. Wang, L. Weitz, D. Wimmer, M. Xiao, C. Yan, P. Ye, Q. Zha, U. Baltensperger, J. Curtius, J. Dommen, R. C. Flagan, M. Kulmala, J. N. Smith, D. R. Worsnop, A. Hansel, N. M. Donahue, P. M. Winkler, Rapid growth of organic aerosol nanoparticles over a wide tropospheric temperature range. *Proc. Natl. Acad. Sci. U.S.A.* **115**, 9122–9127 (2018).

63. G. Chen, F. Canonaco, A. Tobler, W. Aas, A. Alastuey, J. Allan, S. Atabakhsh, M. Aurela, U. Baltensperger, A. Bougiatioti, J. F. De Brito, D. Ceburnis, B. Chazeau, H. Chebaicheb, K. R. Daellenbach, M. Ehn, I. El Haddad, K. Eleftheriadis, O. Favez, H. Flentje, A. Font, K. Fossum, E. Freney, M. Gini, D. C. Green, L. Heikkinen, H. Herrmann, A.-C. Kalogridis, H. Keernik, R. Lhotka, C. Lin, C. Lunder, M. Maasikmets, M. I. Manousakas, N. Marchand, C. Marin, L. Marmureanu, N. Mihalopoulos, G. Močnik, J. Nęcki, C. O'Dowd, J. Ovadnevaite, T. Peter, J.-E. Petit, M. Pikridas, S. Matthew Platt, P. Pokorná, L. Poulain, M. Priestman, V. Riffault, M. Rinaldi, K. Różański, J. Schwarz, J. Sciare, L. Simon, A. Skiba, J. G. Slowik, Y. Sosedova, I. Stavroulas, K. Styszko, E. Teinemaa, H. Timonen, A. Tremper, J. Vasilescu, M. Via, P. Vodička, A. Wiedensohler, O. Zografou, M. Cruz Minguillón, A. S. H. Prévôt, European aerosol phenomenology − 8: Harmonised source apportionment of organic aerosol using 22 Year-long ACSM/AMS datasets. *Environ. Int.* **166**, 107325 (2022).

64. W. R. Leaitch, L. M. Russell, J. Liu, F. Kolonjari, D. Toom, L. Huang, S. Sharma, A. Chivulescu, D. Veber, W. Zhang, Organic functional groups in the submicron aerosol at 82.5°N, 62.5°W from 2012 to 2014. *Atmos. Chem. Phys.* **18**, 3269–3287 (2018).

65. I. El Haddad, N. Marchand, B. D'Anna, J. L. Jaffrezo, H. Wortham, Functional group composition of organic aerosol from combustion emissions and secondary processes at two contrasted urban environments. *Atmos. Environ.* **75**, 308–320 (2013).

66. M. Surdu, J. Top, B. Yang, J. Zhang, J. G. Slowik, A. S. H. Prévôt, D. S. Wang, I. el Haddad, D. M. Bell, Real-time identification of aerosol-phase carboxylic acid production using extractive electrospray ionization mass spectrometry. *Environ. Sci. Technol.* **58**, 8857–8866 (2024).

67. V. Moschos, K. Dzepina, D. Bhattu, H. Lamkaddam, R. Casotto, K. R. Daellenbach, F. Canonaco, P. Rai, W. Aas, S. Becagli, G. Calzolai, K. Eleftheriadis, C. E. Moffett, J.

Schnelle-Kreis, M. Severi, S. Sharma, H. Skov, M. Vestenius, W. Zhang, H. Hakola, H. Hellén, L. Huang, J.-L. Jaffrezo, A. Massling, J. K. Nøjgaard, T. Petäjä, O. Popovicheva, R. J. Sheesley, R. Traversi, K. E. Yttri, J. Schmale, A. S. H. Prévôt, U. Baltensperger, I. El Haddad, Equal abundance of summertime natural and wintertime anthropogenic Arctic organic aerosols. *Nat. Geosci.* **15**, 196–202 (2022).

68. Y. M. Qin, H. B. Tan, Y. J. Li, M. I. Schurman, F. Li, F. Canonaco, A. S. H. Prévôt, C. K. Chan, Impacts of traffic emissions on atmospheric particulate nitrate and organics at a downwind site on the periphery of Guangzhou, China. *Atmos. Chem. Phys.* **17**, 10245–10258 (2017).

69. R. Pohorsky, A. Baccarini, J. Tolu, L. H. E. Winkel, J. Schmale, Modular Multiplatform Compatible Air Measurement System (MoMuCAMS): A new modular platform for boundary layer aerosol and trace gas vertical measurements in extreme environments. *Atmos. Meas. Tech.* **17**, 731–754 (2024).

70. J. B. Pernov, D. Beddows, D. C. Thomas, M. Dall'Osto, R. M. Harrison, J. Schmale, H. Skov, A. Massling, Increased aerosol concentrations in the High Arctic attributable to changing atmospheric transport patterns. *npj Clim. Atmos. Sci.* **5**, 62 (2022).

71. A. Stohl, Characteristics of atmospheric transport into the Arctic troposphere. *J. Geophys. Res. Atmos.* **111**, D11306 (2006).

72. P. K. Quinn, G. Shaw, E. Andrews, E. G. Dutton, T. Ruoho-Airola, S. L. Gong, Arctic haze: Current trends and knowledge gaps. *Tellus B* **59**, 99–114 (2022).

73. M. D. Willis, H. Bozem, D. Kunkel, A. K. Y. Lee, H. Schulz, J. Burkart, A. A. Aliabadi, A. B. Herber, W. R. Leaitch, J. P. D. Abbatt, Aircraft-based measurements of High Arctic springtime aerosol show evidence for vertically varying sources, transport and composition. *Atmos. Chem. Phys.* **19**, 57–76 (2019).

74. E. Schneider, H. Czech, O. Popovicheva, M. Chichaeva, V. Kobelev, N. Kasimov, T. Minkina, C. P. Rüger, R. Zimmermann, Mass spectrometric analysis of unprecedented high levels of carbonaceous aerosol particles long-range transported from wildfires in the Siberian Arctic. *Atmos. Chem. Phys.* **24**, 553–576 (2024).

75. M. D. Willis, W. R. Leaitch, J. P. D. Abbatt, Processes controlling the composition and abundance of arctic aerosol. *Rev. Geophys.* **56**, 621–671 (2018).

76. L. Zamora, G. Sotiropoulou, G. de Boer, R. Calmer, J.-C. Raut, I. Wadlow, Future directions for aerosol–cloud–precipitation interaction research in the arctic from the QuIESCENT 2024 workshop. *Bull. Am. Meteorol. Soc.* **106**, E829–E835 (2025).

77. J. Schmale, P. Zieger, A. M. L. Ekman, Aerosols in current and future Arctic climate. *Nat. Clim. Change* **11**, 95–105 (2021).

78. S. Schmid, M. Kurek, A. Boisen, "Towards airborne nanoparticle mass spectrometry with nanomechanical string resonators," in *Micro- and Nanotechnology Sensors, Systems, and Applications V* (SPIE, 2013), vol. 8725, pp. 414–421; www.spiedigitallibrary.org/conference-proceedings-of-spie/8725/872525/Towards-airborne-nanoparticle-mass-spectrometry-with-nanomechanical-string-resonators/10.1117/12.2013682.full.

79. X. Liu, X. Zhang, J. Schnelle-Kreis, G. Jakobi, X. Cao, J. Cyrys, L. Yang, B. Schloter-Hai, G. Abbaszade, J. Orasche, M. Khedr, M. Kowalski, M. Hank, R. Zimmermann, Spatiotemporal characteristics and driving factors of black carbon in Augsburg, Germany: Combination of mobile monitoring and street view images. *Environ. Sci. Technol.* **55**, 160–168 (2021).

80. H. Bešić, A. Demir, J. Steurer, N. Luhmann, S. Schmid, Schemes for tracking resonance frequency for micro- and nanomechanical resonators. *Phys. Rev. Appl.* **20**, 024023 (2023).

81. H. Bešić, A. Demir, V. Vukićević, J. Steurer, S. Schmid, Adaptable frequency counter with phase filtering for resonance frequency monitoring in nanomechanical sensing. *IEEE Sensors J.* **24**, 8094–8104 (2024).

82. J. Timarac-Popović, J. Hiesberger, E. Šesto, N. Luhmann, A. Giesriegl, A. Bešić, J. P. Lafleur, S. Schmid, Picogram-level nanoplastic analysis with nanoelectromechanical system Fourier transform infrared spectroscopy: NEMS-FTIR. *ACS Nano*, 10.1021/acsnano.5c22099 (2026).

83. M. Piller, J. Hiesberger, E. Wistrela, P. Martini, N. Luhmann, S. Schmid, Thermal IR detection with nanoelectromechanical silicon nitride trampoline resonators. *IEEE Sens. J.* **23**, 1066–1071 (2023).

84. S. Niyogi, E. Bekyarova, M. E. Itkis, J. L. McWilliams, M. A. Hamon, R. C. Haddon, Solution properties of graphite and graphene. *J. Am. Chem. Soc.* **128**, 7720–7721 (2006).

85. D. A. Parks, P. R. Griffiths, A. T. Weakley, A. L. Miller, Quantifying elemental and organic carbon in diesel particulate matter by mid-infrared spectrometry. *Aerosol Sci. Tech.* **55**, 1014–1027 (2021).

86. M. R. Querry, "Optical constants of minerals and other materials from the millimeter to the ultraviolet" (CRDEC-CR-88009, 1987); https://apps.dtic.mil/sti/citations/ADA192210.

87. J. Ren, L. Chen, J. Liu, F. Zhang, The density of ambient black carbon retrieved by a new method: Implications for cloud condensation nuclei prediction. *Atmos. Chem. Phys.* **23**, 4327–4342 (2023).

88. A. Kuzmiakova, A. M. Dillner, S. Takahama, An automated baseline correction protocol for infrared spectra of atmospheric aerosols collected on polytetrafluoroethylene (Teflon) filters. *Atmos. Meas. Tech.* **9**, 2615–2631 (2016).

89. M. Reggente, R. Höhn, S. Takahama, An open platform for Aerosol InfraRed Spectroscopy analysis – AIRSpec. *Atmos. Meas. Tech.* **12**, 2313–2329 (2019).

90. M. Sprenger, H. Wernli, The LAGRANTO Lagrangian analysis tool – version 2.0. *Geosci. Model Dev.* **8**, 2569–2586 (2015).

91. Z. Klimont, K. Kupiainen, C. Heyes, P. Purohit, J. Cofala, P. Rafaj, J. Borken-Kleefeld, W. Schöpp, Global anthropogenic emissions of particulate matter including black carbon. *Atmos. Chem. Phys.* **17**, 8681–8723 (2017).

92. C. D. Elvidge, M. Zhizhin, K. Baugh, F.-C. Hsu, T. Ghosh, Methods for global survey of natural gas flaring from visible infrared imaging radiometer suite data. *Energies* **9**, 14 (2016).

93. G. Baffou, R. Quidant, Thermo-plasmonics: Using metallic nanostructures as nano-sources of heat. *Laser Photonics Rev.* **7**, 171–187 (2013).
